# Effects of non-health-targeted policies on migrant health: a systematic review and meta-analysis

**DOI:** 10.1016/S2214-109X(18)30560-6

**Published:** 2019-03-06

**Authors:** Sol Pía Juárez, Helena Honkaniemi, Andrea C Dunlavy, Robert W Aldridge, Mauricio L Barreto, Srinivasa Vittal Katikireddi, Mikael Rostila

**Affiliations:** a Department of Public Health Sciences, Stockholm University, Stockholm, Sweden; b Centre for Health Equity Studies, Stockholm University/Karolinska Institutet, Stockholm, Sweden; c Centre for Public Health Data Science, Institute of Health Informatics, University College London, London, UK; d The Farr Institute of Health Informatics Research, University College London, London, UK; e Centre for Data and Knowledge Integration for Health, Fiocruz, Salvador, Brazil; f MRC/CSO Social and Public Health Sciences Unit, University of Glasgow, Glasgow, UK

## Abstract

**Background:**

Government policies can strongly influence migrants' health. Using a Health in All Policies approach, we systematically reviewed evidence on the impact of public policies outside of the health-care system on migrant health.

**Methods:**

We searched the PubMed, Embase, and Web of Science databases from Jan 1, 2000, to Sept 1, 2017, for quantitative studies comparing the health effects of non-health-targeted public policies on migrants with those on a relevant comparison population. We searched for articles written in English, Swedish, Danish, Norwegian, Finnish, French, Spanish, or Portuguese. Qualitative studies and grey literature were excluded. We evaluated policy effects by migration stage (entry, integration, and exit) and by health outcome using narrative synthesis (all included studies) and random-effects meta-analysis (all studies whose results were amenable to statistical pooling). We summarised meta-analysis outcomes as standardised mean difference (SMD, 95% CI) or odds ratio (OR, 95% CI). To assess certainty, we created tables containing a summary of the findings according to the Grading of Recommendations Assessment, Development, and Evaluation. Our study was registered with PROSPERO, number CRD42017076104.

**Findings:**

We identified 43 243 potentially eligible records. 46 articles were narratively synthesised and 19 contributed to the meta-analysis. All studies were published in high-income countries and examined policies of entry (nine articles) and integration (37 articles). Restrictive entry policies (eg, temporary visa status, detention) were associated with poor mental health (SMD 0·44, 95% CI 0·13–0·75; *I*^2^=92·1%). In the integration phase, restrictive policies in general, and specifically regarding welfare eligibility and documentation requirements, were found to increase odds of poor self-rated health (OR 1·67, 95% CI 1·35–1·98; *I*^2^=82·0%) and mortality (1·38, 1·10–1·65; *I*^2^=98·9%). Restricted eligibility for welfare support decreased the odds of general health-care service use (0·92, 0·85–0·98; *I*^2^=0·0%), but did not reduce public health insurance coverage (0·89, 0·71–1·07; *I*^2^=99·4%), nor markedly affect proportions of people without health insurance (1·06, 0·90–1·21; *I*^2^=54·9%).

**Interpretation:**

Restrictive entry and integration policies are linked to poor migrant health outcomes in high-income countries. Efforts to improve the health of migrants would benefit from adopting a Health in All Policies perspective.

**Funding:**

Swedish Council for Health, Working Life, and Social Research; UK Medical Research Council; Scottish Government Chief Scientist Office.

## Introduction

WHO's Health in All Policies approach^1^ to improving population health advocates for consideration of the health implications of public policies across all sectors. This perspective is particularly relevant for the health of migrant populations, who are affected by both general and migrant-specific policies in the destination country. Migrant-specific policies include those pertaining to entry (visa and entry criteria), resettlement (dispersal policies), short-term integration (language classes), long-term integration (anti-discriminatory policies in the labour market, democratic participation, and citizenship policies), and forced and voluntary return migration (deportation procedures). More importantly, the steady increase in international migration, from approximately 155 million migrants in 2000, to 258 million in 2017,^2^ has been met with increasingly hostile migration policies worldwide, from expanded EU border control efforts^3^ to attempts to rescind legal protection granted to undocumented migrants under the US Deferred Action for Childhood Arrivals programme.^4^ Although such policies have not been designed to influence migrant health, their role as social determinants of health is incontestable. Previous systematic reviews have examined the health effects of detention policies^5^, ^6^ and documentation requirements^7^ among specific migrant populations, but have not isolated policy effects with comparator populations or examined policies at multiple stages of the migration process. The aim of this systematic review and meta-analysis was to comprehensively examine the effect of non-health-targeted policies on migrant health.

## Methods

### Search strategy and selection criteria

For this systematic review and meta-analysis we searched the PubMed, Embase, and Web of Science databases in September, 2017, for peer-reviewed studies published between Jan 1, 2000, and Sept 1, 2017, and written in English, Swedish, Danish, Norwegian, Finnish, French, Spanish, or Portuguese. Search strings were constructed in collaboration with a medical librarian (appendix). Studies were eligible for inclusion if an international migrant population was studied (as per the International Organization for Migration definition);^8^ a policy intervention, not primarily introduced to improve health, was implemented at the supranational, national, or local level; a relevant comparison group was used (ie, between intervention and control groups, between countries or regions, or before and after policy implementation within a target population); and a health outcome was assessed. All quantitative and mixed-methods designs were considered. Qualitative studies were excluded as our aim was to quantify policy health effects. We did not search grey literature sources. Forward and backward citation searching (ie, identification of newer publications citing a reference included in our review and previous publications cited within an included study) and consultations with topic experts were used in parallel to identify additional articles for inclusion. Article titles and abstracts were imported into the Covidence systematic-review online management tool. Review team members (SPJ, HH, and ACD) alternated review duties for each paper. Two reviewers independently screened titles and abstracts, retrieved full texts of potentially relevant articles, and assessed article eligibility for inclusion, while discrepancies were resolved by a third reviewer.

**Research in context**

**Evidence before this study**

The extent to which health policies are effective in influencing the health of migrants and natives alike has been investigated, yet evidence on the health effects of other public policies among migrants is scarce. To prepare for this systematic review and meta-analysis we used expert recommendations and searched the PubMed and Embase databases from Jan 1, 2000, to July 15, 2017, for English-language review articles that examined the impact of various public policies on the health of migrants. Search terms included “policy”, “migrants”, “refugees”, and “asylum seekers”. We identified three relevant systematic reviews, all published within the last decade, which found increased odds of poor mental health for asylum seekers exposed to detention and undocumented migrants exposed to restrictive (anti-immigrant) policy in the integration phase. Policies that instituted strict documentation requirements were shown to reduce migrant health-care access. However, these reviews considered few aspects of public policy outside of the health-care sector, often did not assess the quality of included studies, and did not assess health among people not exposed to such policies as a comparison group. Previous narrative reviews have highlighted the importance of considering the social determinants of health among migrants.

**Added value of this study**

This systematic review and meta-analysis provides a comprehensive overview of the impact of public policies on a range of migrant health outcomes. All peer-reviewed evidence came from high-income countries and primarily focused on integration policies, with scarce evidence of the effect of entry or exit policies on migrant health. Our findings highlight a mental health disadvantage in refugees with temporary residence protection and stringent reception upon arrival; decreased health-insurance enrolment and health-service use stemming from restrictive welfare policies; and poor self-rated health and psychological health among non-citizen migrants due to documentation-related and general integration-related fears.

**Implications of all the available evidence**

Migration policies are key social determinants of health and can affect health directly, through access to care, and indirectly, through social and economic pathways. This review highlights the capacity of policy mechanisms to affect migrant health negatively and perpetuate migrant health inequities. These effects have yet to be studied in low-income and middle-income contexts and across other dimensions of migration policy, such as educational and housing opportunities and deportation and exit procedures. Based on our findings, we recommend the use of a Health in All Policies approach, which considers the health effects of all migrant-oriented policies. More inclusive approaches towards integration of migrants into their host societies, rather than the enforcement of stringent reception and border control efforts, is likely to have a positive effect on migrants' health and life opportunities. Such policies are also more in line with the spirit of international agreements made to respect and uphold universal human rights.

### Statistical analysis

Review team members (SPJ, HH, and ACD) extracted the data and assessed risk of bias of included papers. Extracted data included sample characteristics of exposure and control groups (ie, sample size, participants' age, gender, origin, and reason for migration), policy measures, health outcomes, control variables, details of the analytical approaches used in studies, types of outcome measures, and measures of association and statistical significance (data extraction sheet available upon request from authors). Risk of bias was assessed with a modified version of the Effective Public Health Practice Project quality assessment tool (appendix).^9^, ^10^ Authors were contacted if additional study information was required.

Our analytical approach entailed narrative synthesis of all included studies and meta-analysis of poolable estimates. Narrative synthesis was done according to the Economic and Social Research Council guidelines.^11^ We categorised policies according to the Migration Phases Framework,^12^ focusing on the entry, integration, and exit phases. Policies were further classified by generosity—ie, whether migrants' access to health-promoting resources and opportunities was increased (generous) or limited (restrictive) by the policy.^13^ Tabulation and modified vote-counting (ie, assessing frequency and direction of effects across studies while accounting for risk of bias) was used as per the Economic and Social Research Council guidance^11^, ^14^ and was paired with subanalyses to explore the heterogeneity of health effects by migrant population and context, when possible.

We did the random-effects meta-analysis by policy domain and health outcome when results were amenable to statistical pooling,^15^ using the metan command in Stata version 13.^16^ Conceptually similar health outcomes assessed with different measures (eg, different indicators of mental health) were meta-analysed together to quantify policy effects on broad categories of health. We grouped outcomes based on whether they were originally reported as dichotomous, which we summarised as odds ratios (ORs; 95% CI), or continuous, which we summarised as standardised mean differences (SMDs; 95% CI). Some measures were dichotomised or transformed from risk ratios to ORs to facilitate inclusion in meta-analysis.^17^ Results were displayed in Forest plots with *I*^2^ statistics to indicate the proportion of true heterogeneity between studies from the total observed variation.^18^ We used the Grading of Recommendations Assessment, Development, and Evaluation (GRADE) approach to summarise and assess the certainty of findings concerning the direction of the effect by outcome.^19^, ^20^ Sensitivity analyses were done to test the robustness of overall findings by excluding studies with high risk of bias,^18^ and to explore heterogeneity of health effects by excluding specific migrant populations (eg, older migrants [ie, aged ≥65 years], child migrants, non-citizen migrants, and undocumented migrants).

Our study was registered with PROSPERO, number CRD42017076104.

### Role of the funding source

The funders of the study had no role in the study design, data collection, data analysis, data interpretation, or writing of the report. All authors had full access to all the data in the study and had final responsibility for the decision to submit for publication.

## Results

**Figure 1:**
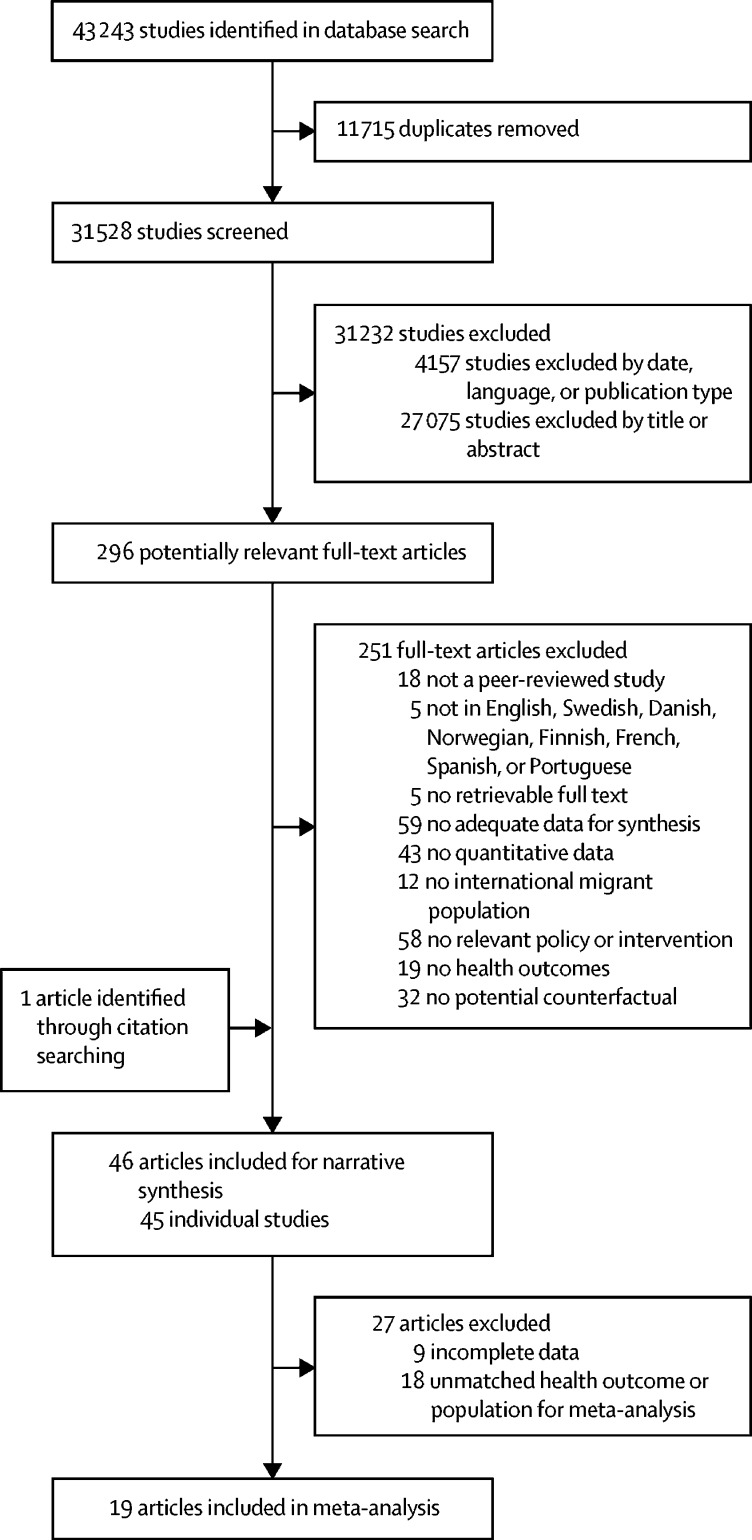
Article selection process

The database search yielded 43 243 records. After the removal of 11 715 duplicates, 31 232 records were excluded by date, language, or publication type, or on the basis of their title or abstract. Of the remaining 296 potentially relevant full-text articles, 251 did not meet the eligibility criteria. One record was included through citation searching. A final 46 articles were included in the narrative synthesis,^21^, ^22^, ^23^, ^24^, ^25^, ^26^, ^27^, ^28^, ^29^, ^30^, ^31^, ^32^, ^33^, ^34^, ^35^, ^36^, ^37^, ^38^, ^39^, ^40^, ^41^, ^42^, ^43^, ^44^, ^45^, ^46^, ^47^, ^48^, ^49^, ^50^, ^51^, ^52^, ^53^, ^54^, ^55^, ^56^, ^57^, ^58^, ^59^, ^60^, ^61^, ^62^, ^63^, ^64^, ^65^, ^66^ of which two reported on the same study,^48^, ^58^ and 19 qualified for meta-analysis^22^, ^25^, ^29^, ^30^, ^34^, ^35^, ^36^, ^37^, ^41^, ^45^, ^47^, ^48^, ^49^, ^50^, ^51^, ^53^, ^60^, ^62^, ^65^ (figure 1; table 1; appendix). All included studies were done in high-income contexts, and examined the health-related impact of entry (eight studies)^32^, ^35^, ^47^, ^48^, ^53^, ^57^, ^58^, ^59^, ^61^ and integration (37 studies)^21^, ^22^, ^23^, ^24^, ^25^, ^26^, ^27^, ^28^, ^29^, ^30^, ^31^, ^33^, ^34^, ^36^, ^37^, ^38^, ^39^, ^40^, ^41^, ^42^, ^43^, ^44^, ^45^, ^46^, ^49^, ^50^, ^51^, ^52^, ^54^, ^55^, ^56^, ^60^, ^62^, ^63^, ^64^, ^65^, ^66^ policies (table 1); no study investigated exit policies. Most studies pertaining to entry had high risk of bias, while integration-related studies generally had low and moderate risks of bias. Findings from the meta-analysis and narrative synthesis are described by policy type (entry or integration), then by health outcome (table 2).

**Table 1:** Descriptive overview of 46 included articles (using data from 45 studies)

	**Country**	**Data source**	**Data years**	**Study design**	**Population group**	**Policy**	**Health outcome**
**Entry policies**							
Goosen et al, 2014^32^	Netherlands	Community Health Services for Asylum Seekers (electronic medical-records database)	2000–08	Cohort	Accompanied asylum seeker children (aged 4–17 years)	Annual relocation rate between asylum-seeker centres	Mental distress (ICPC codes)
Johnston et al, 2009^35^	Australia	Surveys	2004–05	Cross-sectional	Adult Iraqi refugees with TPVs *vs* PPVs	TPV policy (1999–2008): introduced distinction between TPV and PPV; TPV recipients were excluded from or given reduced access to government-funded benefits and services, barred from family reunion programmes, and had mandatory detention from 1992–2008; PPVs were granted to people entering via offshore humanitarian programmes, guaranteed immediate refugee status, and entitled to all services provided to permanent residents	Access to health care and medications; general and physical health (SF-36); psychological distress and depression (HSCL-25); PWI
Miranda et al, 2011^47^	USA	H-EPESE	1993–94	Cross-sectional	Mexican-born migrants (aged ≥65 years)	Sociopolitical context of entry: Post-Mexican Revolution Era (1918–28)—lenient migration policies; Era of Variable Deportations (1929–41)—increased deportation due to domestic labour concerns, variable enforcement of related policies; the Bracero Era (1942–64)—extensive employment of Mexican migrants on farms and railroad projects (Bracero Program); Post-immigration Reform and Control Act Era (1965–94)—increased deportation of most migrants, with social protection of remaining Bracero participants	Depression (CES-D-8)
Momartin et al, 2006^48^*	Australia	STARTTS	2002–03; 2004–05	Cross-sectional	TPV-holding *vs* PPV-holding Persian-speaking asylum seekers	TPV policy (1999–2008; see description for Johnston et al, 2009)	PTSD (HTQ); distress (GHQ-30); depression and anxiety (HSCL-25); functional impairment (SF-12, mental component); excessive worry (PSWQ)
Steel et al, 2011^58^*	Australia	Early Intervention Program of the STARTTS	2002–03; 2004–05	Cohort analytic	TPV-*vs* PPV-holding Persian-speaking asylum seekers	TPV policy (1999–2008; see description for Johnston et al, 2009)	PTSD (HTQ); distress (GHQ-30); depression and anxiety (HSCL-25); functional impairment (SF-12, mental component); excessive worry (PSWQ)
Reijneveld et al, 2005^53^	Netherlands	Psychological assessments and interviews	2002–03	Cross-sectional	Unaccompanied adolescent asylum seekers	Unaccompanied adolescent asylum seekers' reception policies: routine (up to November, 2002); campus (November, 2002, to January, 2005)	Mental health, depression, and anxiety (HSCL-25); PTSD (RATS Inventory)
Steel et al, 2006^57^	Australia	Surveys	Not reported	Cross-sectional	TPV-holding *vs* PPV-holding Arabic-speaking Mandaean refugees	TPV policy (1999–2008; see description for Johnston et al, 2009)	Access to health care and long-term health problems (PMLD); PTSD (HTQ); depression and anxiety (HSCL-25); mental health status and disability (SF-12)
Tan et al, 2016^59^	70 refugee sites in 17 countries	UNHCR Health Information System	2011–12	Cross-sectional	Refugees	UNHCR expenditure on refugee programmes in 2011	Crude and under-5 mortality
Urquia et al, 2015^61^	Canada and Spain	Canada—perinatal surveillance system, immigration register, citizenship and immigration and discharge-abstracts databases; Spain—anonymous birth certificate database	Canada (2000–05); Spain (1998–2007)	Cross-sectional	Singleton births to Latin American-born *vs* Canadian or Spanish native-born mothers	Immigrant entry policies: Canada (restrictive)—favouring labour migration through point system, with fewer refugees and family-class migrants; Spain (less restrictive)—unauthorised migration due to demand for low skill labour and poor management of migration process	Mean birthweight at term; low birthweight; preterm birth
**Integration policies (general)**							
Borrell et al, 2015^26^	18 European countries	ESS	2012	Cross-sectional	Migrants from low-income countries (aged ≥15 years)	National immigrant integration policies—integration country categories based on the MIPEX score†	Association of perceived group discrimination with self-reported health, depression (CES-D, short version), and limitation of activity
Giannoni et al, 2016^31^	23 European countries	EU-SILC	2012	Cross-sectional	Non-European *vs* European migrants or native-born people (aged 16–80 years)	National immigrant integration policies (see description for Borrell et al, 2015)	Self-reported health; limiting long-standing illness; chronic illness
Ikram et al, 2015^34^	3 European countries	Migrant Ethnic Health Observatory (MEHO) Project (linked and unlinked national register data)	Netherlands (1996–2006); France (2005–07); Denmark (1992–2001)	Cohort	Turkish and Moroccan immigrants	National immigrant integration policies (see description for Borrell et al, 2015)	All-cause and cause-specific mortality
Levecque and Van Rossem, 2015^42^	20 European countries	ESS	2006–07	Cross-sectional	Foreign-born *vs* native-born (aged ≥15 years)	National immigrant integration policies—MIPEX scores†	Depression (CES-D-8)
Malmusi, 2015^45^	14 European countries	EU-SILC	2011	Cross-sectional	Non-EU migrants *vs* native-born people (aged ≥16 years)	National immigrant integration policies (see description for Borrell et al, 2015)	Self-reported health
Malmusi et al, 2017^46^	17 European countries	ESS	2012	Cross-sectional	Migrants from low-income countries vs native-born people (aged ≥15 years)	National immigrant integration policies (see description for Borrell et al, 2015)	Depression (CES-D-8)
**Integration policies (welfare)**							
Angus, DeVoe, 2010^23^	USA	Family planning service data	2005–08	Controlled before–after	Hispanic *vs* non-Hispanic family planning-service users (aged ≥18 years)	DRA (2005): required states to collect proof of citizenship to qualify or reapply for federal matching funds for services provided to Medicaid recipients	Family planning service visits
Borjas, 2003^25^	USA	CPS, March Supplement	1995–96; 1999–2001	Controlled before–after	Non-citizens, naturalised citizens *vs* natives (aged <65 years); children (aged <18 years)	PRWORA (1996): modified welfare eligibility rules and prohibited states from using federal funds for newly unqualified immigrant recipients (arriving in the USA after August, 1996, for the first five years of settlement), including changes to public health insurance (Medicaid) coverage for non-emergency services	Health insurance coverage (Medicaid, any type, employer-sponsored)
Bozorgmehr and Razum, 2015^27^	Germany	German Federal Statistics Office	1994–97; 1997–2007; 2007–13	Controlled before–after	Asylum seekers and refugees (*vs* people with regular welfare access)	Asylum Seekers' Benefits Act (1993) and reforms (1997, 2007): expanded waiting time for access to welfare services for asylum seekers and refugees from 12 to 36 months (reform 1; 1997) and 48 months (reform 2; 2007)	Health expenditures
Cho, 2011^28^	USA	National Centre for Health Statistics	1995–96; 1999–2002	Controlled before–after	Foreign-born *vs* US-born low-educated women of Mexican origin	PRWORA (1996; see description for Borjas, 2003)	Infant mortality rate
Fritsch, 2011^29^	USA	CPS, March supplement	2005, 2007	Controlled before–after	Non-citizens *vs* citizens	DRA (2005; see description for Angus & Devoe, 2010)	Health insurance coverage (Medicaid)
Fuentes-Afflick et al, 2006^30^	USA	Structured interviews	1999–2001	Cross-sectional	Foreign-born *vs* US-born Hispanic mothers in California, Florida, and New York	PRWORA (1996; see description for Borjas, 2003)	Use of prenatal care
Joyce et al, 2001^36^	USA	Birth files	1995; 1998	Cohort analytic	Foreign-born *vs* US-born Latina mothers in California, New York City, and Texas	PRWORA (1996; see description for Borjas, 2003)	Financing of care (insured *vs* uninsured or self-pay); early initiation of prenatal care; proportion of low-birthweight births
Kandula et al, 2004^37^	USA	CPS, March Supplement	1994–96; 1998–2001	Controlled before–after	Qualified immigrant *vs* US-born low-income participants	PRWORA (1996; see description for Borjas, 2003)	Medicaid enrolment
Kaushal and Kaestner, 2005^38^	USA	CPS, March Supplement	1994–96; 1998–2001	Controlled before–after	Foreign-born, low-educated single mothers; their children	PRWORA (1996; see description for Borjas, 2003); limitations on Temporary Aid to Needy Families	Health insurance coverage (public, private, employer-sponsored, uninsured)
Kaushal and Kaestner 2007^39^	USA	NHIS	1992–96; 1998–2002	Controlled before–after	Foreign-born, low-educated single mothers (aged 18–54 years); their children (aged 0–14 years)	PRWORA (1996; see description for Borjas, 2003)	Health insurance coverage (public, uninsured); medical care utilisation; self-reported health
Korenbrot et al, 2000^41^	USA	Electronic birth-certificate database	1994; 1995; 1996–97	Controlled before–after	All singleton births to foreign-born *vs* US-born mothers	California Proposition 187 (1994)—prohibited state Medicaid coverage of non-emergency pregnancy-related services for unqualified migrants; PRWORA (1996; see description for Borjas, 2003); IIRAIRA (1996)—established higher-income requirements of migrant sponsors, making Medicaid use a potential liability for sponsorship as evidence of lack of sufficient resources	Number of births by prenatal-care coverage; inadequate prenatal-care use (onset, number of visits); birth outcomes (low birthweight, preterm)
Loue et al, 2005^43^	USA	Interviews	1999–2001	Cross-sectional	Women of Mexican origin	PRWORA (1996; see description for Borjas, 2003) IIRAIRA (1996; see description for Korenbrot et al, 2000)	Difficulty receiving care; satisfaction with care
Lurie, 2008^44^	USA	Survey of Income and Program Participation (SIPP)	1996; 2001	Controlled before–after	Children of non-citizen permanent residents *vs* non-permanent residents in select states	PRWORA (1996; see description for Borjas, 2003)	Health insurance coverage (uninsured)
Nam, 2008^49^	USA	CPS, March Supplement	1994–96; 2001–05	Controlled before–after	Non-citizen, naturalised citizen vs native participants (aged ≥65 years)	PRWORA (1996; see description for Borjas, 2003)	Health insurance coverage (Medicaid, private, employer-sponsored, none)
Nam, 2011^50^	USA	CPS, March Supplement	1994–96; 2003–08	Controlled before–after	Immigrant *vs* native participants (aged ≥65 years)	PRWORA (1996; see description for Borjas, 2003)	Health insurance coverage (Medicaid, Medicare, employer-sponsored, direct purchase, any)
Pati and Danagoulian, 2008^51^	USA	NHIS	1997–2000; 2001–04	Controlled before–after	Foreign-born *vs* mixed-status, US-born low-income children	IIRAIRA (1996; see description for Korenbrot et al, 2000); IIRAIRA public charge rule reversal (1999)—Medicaid benefits exempted from IIRAIRA's public charge rule	Health insurance coverage (uninsured, private, public)
Sommers, 2010^56^	USA	CPS, March Supplement	2004–06; 2007–08	Controlled before–after; cohort analytic	Non-citizen *vs* citizen participants; adults (aged 19–64 years); children (aged 0–18 years)	DRA (2005; see description for Angus & Devoe, 2010)	Medicaid or CHIP enrolment; Medicaid retention
Yeo, 2017^65^	USA	NHIS	1993–96; 2002–13	Controlled before-after	Foreign-born *vs* native-born participants (aged ≥65 years)	PRWORA (1996; see description for Borjas, 2003)	Outpatient healthcare service use
Zhu and Xu, 2015^66^	USA	CPS, March Supplement	1998–2010	Time series	Foreign-born vs. native-born participants	PRWORA (1996; see description for Borjas, 2003)	Inequality in Medicaid coverage
**Integration policies (documentation)**							
Amuedo-Dorantes et al, 2013^21^	USA	Unnamed survey	2009–10	Cross-sectional	Deported *vs* voluntarily returning undocumented Mexican immigrants (aged ≥18 years)	E-Verify (1996): online system matching employment eligibility form (I-9) to data from US Government records	Difficulties obtaining health-care services
Anderson and Finch, 2014^22^	USA	BRFSS	2009; 2010–11	Controlled before–after	Hispanic *vs* Caucasian participants (aged 18–99 years)	SB 1070 (2010–12): required migrants to carry documentation at all times, established state law enforcement responsibility to require documentation during “lawful contacts”, and criminalised work for illegal migrants	Self-reported health status
Beniflah et al, 2013^24^	USA	PED charts	2009–10; 2011	Controlled before-after	Hispanic *vs* non-Hispanic paediatric patients (aged <18 years)	Georgia House Bill 87 (2011): granted local law enforcement the authority to enforce immigration laws	Percent PED visits; percent high acuity visits; PED admission rates
Hatzenbuehler et al, 2017^33^	USA	BRFSS	2012	Cross-sectional	Latino *vs* all participants in selected states (aged ≥18 years)	State-level anti-immigrant policies (eg, with regards to mobility, labour or employment, post-secondary education, health, other services, language, omnibus)	Number of poor mental health days per month; psychological distress (K6)
Kim and Lee, 2011^40^	South Korea	Biological data	1997; 2005	Cross-sectional	Male-migrant lead-industry workers	Legal employment permit system for qualified migrants (2003)	Blood lead levels and other lead biomarkers
Patler and Laster Pirtle, 2017^52^	USA	DACA study (phone survey)	2014–15	Cross-sectional	DACA-eligible Latinos (with *vs* without DACA) in California	DACA (2012): 2-year (renewable) prosecutorial discretion for unauthorised migrants with regard to deportation, with work authorisation and access to social-security card; applicable to those who arrived in the USA before turning 16 years of age (and have proof) and were aged <31 years when the programme began	Psychological distress; negative emotions
Rhodes et al, 2015^54^	USA	North Carolina Vital Statistics System	2005–06; 2009	Controlled before–after	Hispanic or Latina *vs* non-Hispanic and non-Latina mothers	Immigration and Nationality Act Section 287 (g) (via IIRAIRA; 1996)—authorised local law-enforcement agencies to enforce federal immigration law by targeting or removing undocumented migrants convicted of various crimes; Secure Communities Program: facilitated sharing of pertinent information on local arrestees to confirm documentation status via immigration databases	Late entry into prenatal care; inadequate prenatal care
Salmasi and Pieroni, 2015^55^	Italy	Birth Sample Survey (Italian Institute of Statistics)	2000–01; 2003	Controlled before–after	Non-citizen *vs* naturalised and native-born Italian mothers	Italian Laws 189/2002 and 222/2002: granted amnesty for illegal immigrant workers and allowed for regularisation of migrants in domestic service (189) and other industries (222)	Low birthweight
Toomey et al, 2004^60^	USA	Ongoing quasi-experimental longitudinal interview study	2007–08; 2008–09; 2009–10; 2010–11	Cohort	Adolescent mothers (aged 15–18 years) of Mexican origin; their children, their mother figures (eg, mother, grandmother, aunt)	SB 1070 (2010–2012; see description for Anderson, Finch, 2014)	Receipt of public assistance; preventive health-care use for self and child
Vargas et al, 2017^62^	USA	Latino National Health and Immigration Survey (LNHIS)	2015	Cross-sectional	Adult Latinos	General perceptions of anti-immigration laws	Self-rated health; problems with mental health
Venkataramani et al, 2017^63^	USA	NHIS	2008–12; 2012–15	Controlled before–after	Non-citizen Hispanic adults (aged 19–50 years)	DACA (2012; see description for Patler, Laster Pirtle, 2017)	Self-rated health; psychological distress (K6)
White et al, 2014^64^	USA	Electronic health records data	2010–12	Time series	Latino *vs* non-Latino health-clinic visits; adults and children	Alabama Taxpayer and Citizen Protection Act (House Bill 56; 2011): required proof of lawful US residence to receive state and local public benefits (including publicly funded health services)	Healthcare clinic service visits

ICPC=International Classification of Primary Care. TPV=Temporary Protection Visa. PPV=Permanent Protection Visa. SF-36=36-Item Short Form Health Survey. HSCL-25=Hopkins Symptom Checklist (25 item). PWI=Personal Wellbeing Index. H-EPESE=Hispanic established populations for the epidemiologic studies of the elderly. CES-D=Centre for Epidemiological Studies Depression. STARTTS=Scale. Service for the Treatment and Rehabilitation of Torture and Trauma Survivors. PTSD=post-traumatic stress disorder. HTQ=Harvard Trauma Questionnaire. GHQ-30=General Health Questionnaire-30. SF-12=12-Item Short Form Health Survey. PSWQ=Penn State Worry Questionnaire. RATS=Reactions of Adolescents to Traumatic Stress Inventory. PMLD=Post Migration Living Difficulties Scale. UNHCR=UN High Commissioner for Refugees. ESS=European Social Survey. MIPEX=Migrant Integration Policy Index. EU-SILC=Eurostat European Union Statistics on Income and Living Conditions. DRA=Deficit Reduction Act. CPS=Current Population Survey. PRWORA=Personal Responsibility and Work Opportunity Reconciliation Act. NHIS=National Health Interview Surveys. IIRAIRA=Illegal Immigration Reform and Immigrant Responsibility Act. CHIP=Children's Health Insurance Program. BRFSS=Behavioural Risk Factor Surveillance System. SB 1070=Arizona's Support Our Law Enforcement and Safe Neighbourhoods Act. PED=paediatric emergency department. K6=Kessler Psychological Distress Scale. DACA=Deferred Action for Childhood Arrivals.

* Momartin et al, 2006^48^ and Steel et al, 2011^58^ describe the same study.

† MIPEX score: inclusive (promotes societal participation and citizenship irrespective of labour market attachment, with cultural and political tolerance); assimilationist (promotes societal participation and citizenship irrespective of labour market attachment, but with emphasis on sociopolitical conformity); and exclusionist (access to welfare support and services are conditional to labour-market attachment, scarce opportunities for citizenship).

**Table 2:** GRADE summary of findings: health outcomes of non-health-targeted policies among migrants

	**Certainty assessment**						**Summary of findings**		
	Participants, n	Study design	Risk of bias	Inconsistency	Indirectness	Imprecision	Health impact	Indicative effect sizes,* SMD, OR, or β (95% CI)	Certainty
**Entry (exposure: restrictive policies pertaining to entry)**									
Mental health^32^, ^38^, ^47^, ^48^, ^53^, ^57^, ^58^	9841	Observational	S (−1)	No	No	No	Negative	SMD 0·44 (0·13 to 0·75)	We have very low certainty that more restrictive policies pertaining to entry increase levels of poor mental health†
**Integration (exposure: restrictive policies pertaining to integration)**									
Self-rated health^22^, ^26^, ^31^, ^35^, ^39^, ^45^, ^62^, ^63^	467 142	Observational	S (−1)	No	No	S (−1)	Negative	Assimilationist: OR 1·31 (1·16 to 1·45); exclusionist: OR 2·39 (1·99 to 2·78)	We have very low certainty that more restrictive policies in the integration phase increase the odds of poor self-rated health‡
Mortality^34^	6 848 961 person-years at risk	Observational	No	No	No	No	Negative	Assimilationist: OR 0·73 (0·58 to 0·89); exclusionist: OR 2·14 (1·71 to 2·57)	We have moderate certainty that more restrictive policies in the integration phase increase the odds of all-cause mortality§
Mental health^33^, ^42^, ^46^, ^52^, ^62^, ^63^	73 571	Observational	No	No	No	S (−1)	Negative	β 0·35 (−0·13 to 0·82);^33^ OR 1·58 (1·03 to 2·42)^62^	We have low certainty that more restrictive policies in the integration phase increase the risk of poor mental health¶
Public health insurance coverage^25^, ^29^, ^37^, ^38^, ^49^, ^50^, ^56^	2 461 984	Observational	No	No	No	S (−1)	Negative	OR 0·89 (0·71 to 1·07)	We have low certainty that more restrictive welfare policies in the integration phase reduced migrant Medicaid coverage‖
Uninsured status^36^, ^38^, ^39^, ^44^, ^49^, ^51^	784 775	Observational	No	No	No	S (−1)	Negative	OR 1·06 (0·90 to 1·21)	We have low certainty that more restrictive welfare policies in the integration phase increased the proportion of uninsured migrants**
General health-care service use^23^, ^24^, ^39^, ^60^, ^64^, ^65^	1 154 912	Observational	S (−1)	No	No	S (−1)	Negative	OR 0·92 (0·85 to 0·98)	We have very low certainty that more restrictive policies in the integration phase decreased service use††

GRADE=Grading of Recommendations Assessment, Development, and Evaluation. SMD=standardised mean difference. OR=odds ratio. β=β coefficient. S (−1)=serious risk of bias, downgrade by one point.

* From meta-analysis, unless otherwise indicated.

† Rating down for study design and risk of bias (severe, not very severe, since some risk accounted for in study design); some inconsistency (eg, Miranda et al, 2011^47^), but with conditional explanations (heterogeneity of groups).

‡ Rating down for study design, risk of bias, and imprecision (non-significant effects in narrative synthesis, depending on policy categorisation).

§ Rating down for study design, up for plausible confounding (causes of mortality, gender, etc); some inconsistency, but with conditional explanations (ie, exclusive, assimilationist).

¶ Rating down for study design and imprecision (non-significant effects, depending on policy categorisation), up for plausible confounding (protective policies show opposite effects).

‖ Rating down for study design and imprecision, up for plausible confounding (state effects, fear of enrolment); some inconsistency, but with conditional explanations (heterogeneity of groups).

** Rating down for study design and imprecision, up for plausible confounding (state effects); some inconsistency, but with conditional explanations (heterogeneity of groups; working-age population more capable of moving from public to employer-sponsored insurance than children and elderly).

†† Rating down for study design and risk of bias (not very severe, since some risk accounted for in study design), and imprecision; some inconsistency, but with conditional explanations (heterogeneity of groups; migrants more likely to seek care for their children); some indirectness (ethnicity as proxy for migration status), but acceptable.

Entry policies were examined in eight studies primarily in relation to mental health,^32^, ^35^, ^47^, ^48^, ^53^, ^57^, ^58^ but also to birth outcomes,^61^ mortality,^59^ and health-care access.^35^, ^57^ Seven studies^35^, ^47^, ^48^, ^53^, ^57^, ^59^, ^61^ were cross-sectional and two^32^, ^58^ had cohort designs. Study contexts included Australia,^35^, ^48^, ^57^, ^58^ the Netherlands,^32^, ^53^ the USA,^47^ comparative studies of Canada and Spain,^61^ and refugee sites in multiple countries.^59^ Most studies examined refugees or asylum seekers, and two^32^, ^53^ specifically studied asylum-seeking children.

**Figure 2:**
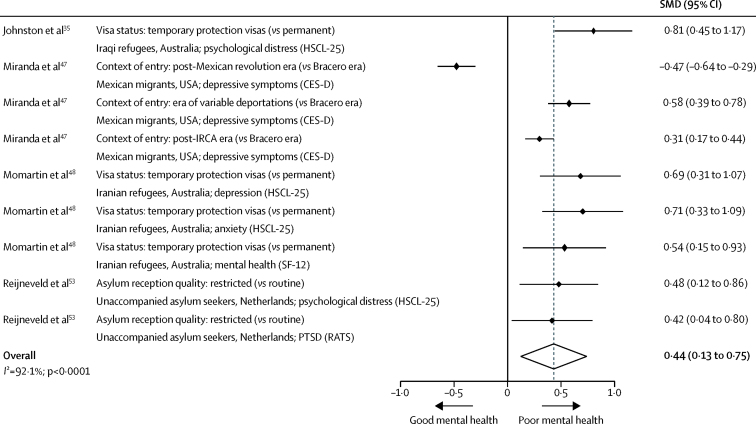
Random-effects meta-analysis of the effects of entry policies on mental health among migrants Figure includes information on policy and comparison; study reference; migrant population and host country; and specific health outcome and measurement instrument. Fully-adjusted estimates were included from each study, with adjustment variables varying by study (data not shown). CES-D=Center for Epidemiological Studies-Depression Scale. HSCL-25=Hopkins Symptom Checklist (25 item). IRCA=Immigration Reform and Control Act. PTSD=post-traumatic stress disorder. RATS=Reactions of Adolescents to Traumatic Stress Inventory. SF-12=Short Form Health Survey (12 item). SMD=standardised mean difference.

Meta-analysis indicated that restrictive entry policies were associated with poor mental health outcomes (ie, psychological distress, depression, anxiety, and post-traumatic stress disorder; SMD 0·44, 95% CI 0·13–0·75, *I*^2^=92·1%; figure 2),^35^, ^47^, ^48^, ^53^ specifically with regard to temporary (*vs* permanent) protection visas, restrictive (*vs* open) border control, and reduced (*vs* freer) mobility in detainment. Despite high risk of bias and heterogeneous policy contexts and analytical methods across most entry policy studies, narrative synthesis largely supported these findings (appendix).^35^, ^47^, ^53^, ^57^, ^58^

Restrictive entry policies were associated with decreased odds of low birthweight among migrants in one study,^61^ perhaps because of positive health selection. However, a high-risk-of-bias study^59^ of forced migration to refugee camps, which might be less likely to be affected by health selection than labour-based migration, revealed decreased crude and under-5 mortality with increased (ie, generous) spending on refugee protection and assistance programmes. Other evidence with high risk of bias showed increased^57^ or similar levels of difficulties^35^ in obtaining access to health care among refugees granted temporary versus permanent protection visas.

The 37 identified studies pertaining to the integration stage of migration were subdivided into categories of general integration, welfare, and documentation policies (table 1). Studies of general integration policies^26^, ^31^, ^34^, ^42^, ^45^, ^46^ considered the broad approaches adopted by different European destination countries, using the Migrant Integration Policy Index (MIPEX) to categorise policy contexts (inclusive, assimilationist, and exclusionist).^67^ Studies on migrant-specific welfare policies in the USA^23^, ^25^, ^28^, ^29^, ^30^, ^36^, ^37^, ^38^, ^39^, ^41^, ^43^, ^44^, ^49^, ^50^, ^51^, ^65^, ^66^ and in Germany^27^ assessed efforts to curtail non-citizen welfare service use (including public health insurance) via waiting times,^25^, ^27^, ^28^, ^30^, ^36^, ^37^, ^38^, ^39^, ^41^, ^43^, ^44^, ^49^, ^50^, ^65^, ^66^ cost barriers,^41^, ^43^, ^51^ and burden of proof for eligibility,^23^, ^29^, ^56^ with controlled before–after and difference-in-difference designs. Finally, studies of documentation policies published in the USA,^21^, ^22^, ^24^, ^33^, ^52^, ^54^, ^60^, ^62^, ^63^, ^64^ Italy,^55^ and South Korea^40^ examined efforts to institute a burden of proof for legal residence, to be used for employment and law-enforcement purposes, while also considering migrants' experiences of fear and discrimination.

**Figure 3:**
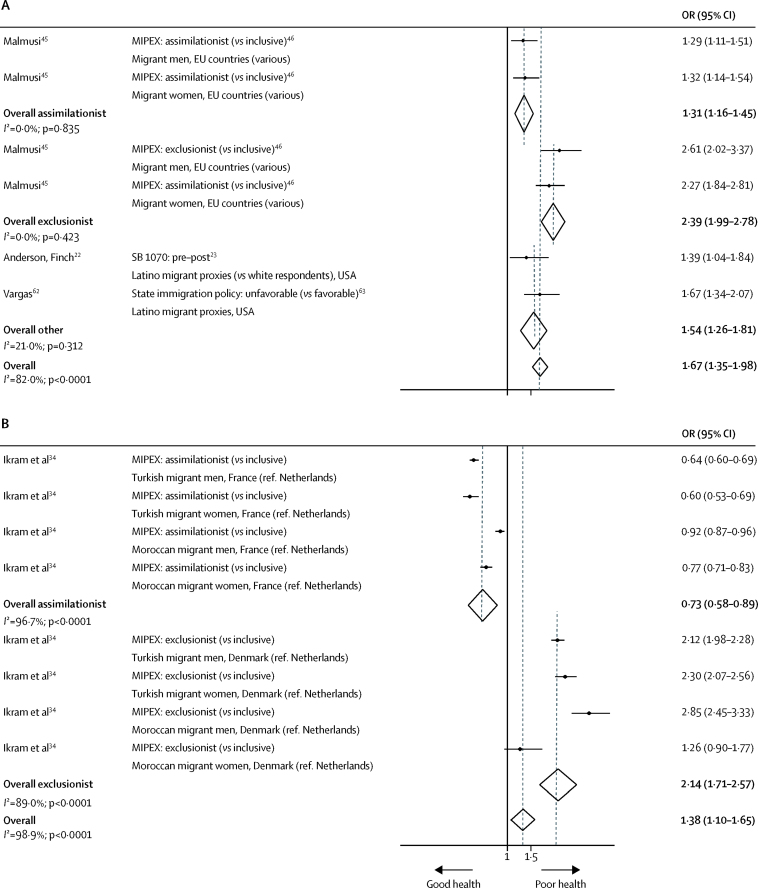
Random-effects meta-analysis of the effects of general-integration and documentation policies on self-rated health (A) and all-cause mortality (B) among migrants Figure includes information on policy and comparison; study reference; migrant population, host country, and population counterfactual (if applicable). Fully-adjusted estimates were included from each study, with adjustment variables varying by study (data not shown). MIPEX=Migrant Integration Policy Index. OR=odds ratio. SB 1070=Arizona's Support Our Law Enforcement and Safe Neighborhoods Act.

Meta-analysis of integration studies indicated that more restrictive policies across the three subcategories were associated with increased odds of poor self-rated health (OR 1·67, 95% CI 1·35–1·98, *I*^2^=82·0%; figure 3a).^22^, ^45^, ^62^ Exclusion of evidence with high risk of bias did not affect this finding (1·68, 1·31–2·05, *I*^2^=84·8%).^62^ Meta-analysis of MIPEX-based general policy approaches found that the odds of poor self-rated health were increased among migrants in assimilationist (1·31, 1·16–1·45, *I*^2^=0·0%) and exclusionist (2·39, 1·99–2·78, *I*^2^=0·0%) contexts relative to inclusive contexts.^45^ Narrative synthesis of studies with low risk of bias supported this gradient of poor self-rated health by increasingly restrictive general policy contexts.^26^, ^31^ However, examination of both welfare and documentation policies revealed mixed associations with self-rated health (appendix).^22^, ^39^, ^62^, ^63^

Similarly, relative to inclusive approaches to integration (as per MIPEX), migrant mortality risks were elevated by more restrictive general policy approaches (OR 1·38, 95% CI 1·10–1·65, *I*^2^=98·9%; figure 3b), specifically in exclusionist settings (2·14, 1·71–2·57, *I*^2^=89·0%), among both men (2·45, 1·74–3·17, *I*^2^=89·4%) and women (1·80, 0·78–2·81, *I*^2^=94·0%).^34^ A decreased risk of all-cause mortality was observed in assimilationist contexts (0·73, 0·58–0·89, *I*^2^=96·7%) and among women specifically (0·69, 0·52–0·85, *I*^2^=91·0%), possibly attributable to variation in health-care access and quality within these policy classifications.^34^

Subcategories of integration policies were found to have mixed effects on mental health. With inadequate data for meta-analysis, narrative synthesis of general integration policies revealed that all migrants had worse health than natives,^42^, ^46^ with the greatest mental health gap in exclusionist contexts, followed by assimilationist, and finally, inclusive contexts (as per the MIPEX score).^46^ Protective documentation policy was shown to safeguard undocumented migrants against poor mental health in robust^63^ and weak^52^ studies alike, while evidence with moderate and high risk of bias for the mental health effects of restrictive documentation policies was mixed.^33^, ^62^

**Figure 4:**
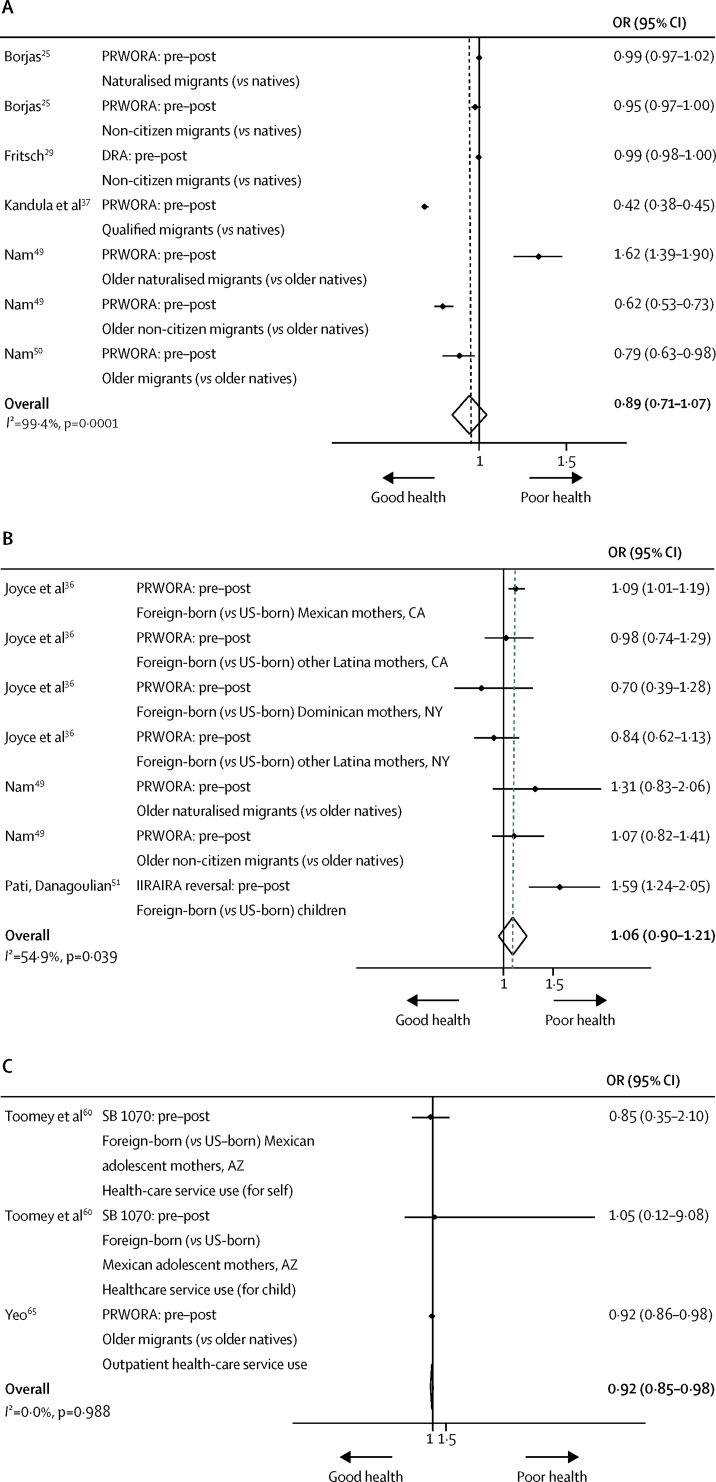

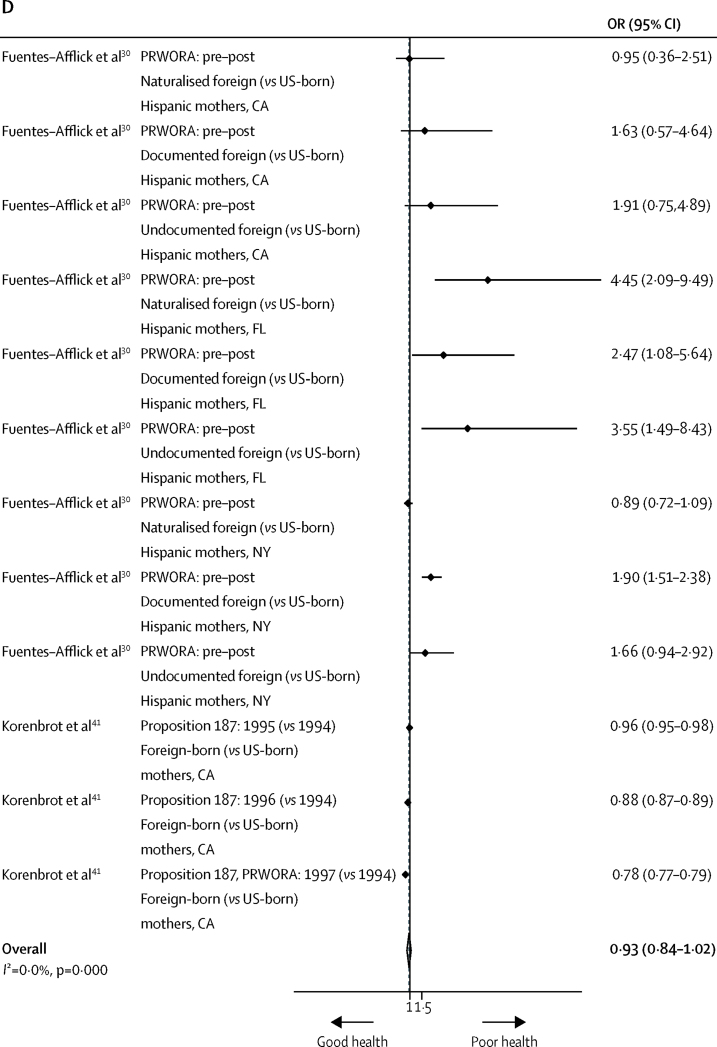
Random-effects meta-analysis of the effects of US welfare restrictions on health-insurance (Medicaid) enrolment (A), the odds of being uninsured (B), health-care service use (C), and prenatal care use (D) among migrants Figure includes information on policy and comparison; study reference; migrant population, US state context, and population counterfactual; and specific health outcome (if applicable). Fully-adjusted estimates were included from each study, with adjustment variables varying by study (data not shown). DRA=Deficit Reduction Act. IIRAIRA=Illegal Immigration Reform and Immigrant Responsibility Act. OR=odds ratio. PRWORA=Personal Responsibility and Work Opportunity Reconciliation Act. SB 1070=Arizona's Support Our Law Enforcement and Safe Neighborhoods Act.

Several studies also assessed the effects of welfare and documentation policy on health insurance and health-care service use. US policies restricting entitlement to welfare support showed no significant reduction in migrants' public health insurance (ie, Medicaid) enrolment (OR 0·89, 95·% CI 0·71–1·07, *I*^2^=99·4%; figure 4A) in the meta-analysis.^25^, ^29^, ^37^, ^49^, ^50^ However, post-hoc analysis that excluded non-working-age migrants showed a reduction in the odds estimate—albeit not significant—for enrolment (0·80, 0·50–1·10, *I*^2^=99·8%), suggesting a potential policy effect for some migrants.^25^, ^29^, ^37^ In line with welfare policies' contingency on citizenship, post-hoc analysis similarly found lower odds of enrolment among non-citizens than among their naturalised peers (0·87, 0·74–1·00, *I*^2^=96·3%).^25^, ^29^, ^49^ Synthesis of studies with low and moderate risk of bias revealed mixed effects of heterogeneous welfare policies on Medicaid enrolment in diverse migrant populations (appendix).^25^, ^29^, ^37^, ^38^, ^49^, ^50^, ^56^, ^66^

Welfare restrictions likewise did not appear to affect odds of being uninsured (ie, lacking any form of health insurance, public or private) as an unqualified migrant relative to qualified individuals (OR 1·06, 95% CI 0·90–1·21, *I*^2^=54·9%; figure 4B), as per our meta-analysis.^36^, ^49^, ^51^ Narrative synthesis of studies with low and moderate risk of bias showed increases in uninsured status for women^38^ and children,^38^, ^44^, ^51^ or no change in insurance status among women,^39^ children,^39^ and older people.^49^ This discrepancy could be attributable to age heterogeneity. Post-hoc meta-analysis excluding children and older migrants revealed that increased welfare restrictions did not lead to working-age migrants becoming uninsured (0·97, 0·80–1·13, *I*^2^=49·7%).^36^ State-level policies in the USA that guaranteed migrants' right to public insurance were mostly unsuccessful in buffering insurance loss relative to states without supplementary insurance,^38^, ^39^ with only one study with moderate risk of bias showing a state-protective effect against insurance loss after welfare reform.^36^

Restrictive welfare policies were also associated with reduced migrant use of health-care services (OR 0·92, 95% CI 0·85–0·98, *I*^2^=0·0%; figure 4C).^60^, ^65^ In the narrative synthesis, studies with low^39^ and greater^23^, ^60^, ^64^, ^65^ risk of bias showed that restrictive welfare and documentation policy discouraged adult,^23^, ^64^ maternal,^39^, ^60^ and older migrant service use,^65^ but not children's use.^39^, ^60^, ^64^ This was perhaps because migrant parents were more likely to seek care for their children than themselves.^64^ A study with high risk of bias on documentation policy also showed decreased service use in paediatric emergency wards, counteracted by increased acute admissions.^24^ This was in line with low-risk-of-bias evidence showing increased societal health expenditures in Germany following the postponement of asylum-seeker and refugee welfare eligibility,^27^ suggesting that restricting health-care access can delay treatment of existing health issues and ultimately force migrants to seek more costly acute care.

However, welfare restrictions did not exert large effects on prenatal health-care service use specifically (OR 0·93, 95% CI 0·84–1·02, *I*^2^=97·6%; figure 4D).^30^, ^41^ Subgroup analyses revealed that odds of inadequate prenatal-care use decreased among protected US states with supplementary welfare packages (ie, CA and NY; 0·92, 0·83–1·01, *I*^2^=98·2%)^30^, ^41^ but increased for unprotected states (ie, FL; 3·14, 1·45–4·84, *I*^2^=0·0%),^30^ from before to after the introduction of federal welfare restrictions. Evidence in the narrative synthesis was mixed with regard to the effects of this state-level welfare policy^30^, ^36^, ^41^, ^43^ and of documentation policies^54^ (appendix), alluding to individual patterns of prenatal-care use dependent on one's nativity and state residency.

For birth outcomes, one study with low risk of bias showed that welfare restrictions were associated with increased infant mortality among Mexican immigrants in the USA compared with natives.^28^ These restrictions did not appear to affect low birthweight (moderate risk of bias),^36^, ^41^ except by state-level differences in supplementary welfare programmes.^36^ Finally, a robust Italian study on documentation policy granting legal rights to previously undocumented women indicated decreased odds of low birthweight in their children.^55^

Given the observational nature of the included studies, our findings are largely reported with low or very low certainty (table 2). More restrictive entry policies were shown to increase levels of poor mental health, albeit with very low certainty given the predominance of studies with high risk of bias. Restrictive policies in the integration phase were associated with decreased self-rated health, once again with very low certainty, given the studies' risks of bias and imprecision (ie, null effects for documentation policies). However, we have moderate certainty that increasingly restrictive general policies of integration increase migrant mortality—inconsistencies in these effects are probably attributable to differences in the contexts and migrant populations studied within each MIPEX integration category. Mental health was shown to worsen with more restrictive policies in the integration phase, a finding we report with low certainty given some imprecision in the estimates. Restrictive welfare policies were associated with decreased public health insurance and increased proportion of uninsured (low certainty). Inconsistencies in these findings appear to be partly attributable to age differences, with working-age migrants more likely to lose public health insurance but less likely to be uninsured overall than children and older migrants. Finally, we have very low certainty that more restrictive policies in the integration phase decreased health-care service use among migrants. Study findings differed by age group, with children's health-care service use less affected than that of adult and older migrants, and by service type, with decreases in primary care use countered by heightened use of acute care.

## Discussion

This systematic review and meta-analysis examined the effect of non-health-targeted public policies on migrant health. The included studies focused exclusively on policies in high-income countries related to the entry and integration phases of the migration journey.

We found evidence for negative effects on mental health in relation to restricted entry, including policies on temporary protection, detention, and restricted asylum reception. Studies also revealed a gradient in health inequalities (poor self-rated health and mortality) between natives and migrants by severity of restrictive policy. Together, these findings suggest that the use of restrictive entry and integration policies should be avoided. If these policies are to be used in exceptional circumstances, they should be implemented alongside strategies to mitigate mental and physical health risks, and to protect the health of refugee migrant groups as per the international responsibilities of nations and states. We also found that increased risks of poor mental health in settings with strict documentation requirements were mirrored by the protective mental health effects of generous documentation policy. This implies that policy makers should not only aim to avoid poor health outcomes by reducing the implementation of harmful policies, but actively work to improve migrant health through the maintenance of generous policy efforts. Finally, there was conflicting evidence that restricting eligibility for welfare support resulted in reduced health insurance coverage. Yet, evidence of policy effects on specific types of health-care use were more consistent, whereby migrants received increased inadequate prenatal care, had reduced perinatal outcomes, and increased the use of acute care services, probably because individuals delayed care for concerns regarding eligibility and affordability. These factors can amplify long-term health-care costs, thus providing an economic incentive for policy revisions. Alternatively, policies ensuring earlier engagement in primary and routine health-care services for migrants would be both health-promoting and cost-mitigating.

To our knowledge, this is the first systematic review and meta-analysis to examine the effects of non-health-targeted public policies on migrant health. Although previous research has found health inequalities among migrants as a result of societal and institutional factors—many of which are affected by public policies^68^—little has been done to isolate the effects of these policies on migrant health. Furthermore, comprehensive evidence on policies across country contexts and migrant populations has been lacking. Our review aimed to summarise research across these dimensions and revealed a strong regional dispersal of research on specific migrant policy domains. The Australian literature largely focused on entry criteria among asylum seekers,^35^, ^48^, ^57^, ^58^ reflecting ongoing international criticism of its detainment procedures.^69^ Most European studies centred on general integration efforts, particularly labour-market participation.^27^, ^31^, ^34^, ^42^, ^45^, ^46^ Research from the USA prioritised documentation status and general attitudes towards migrants,^21^, ^22^, ^24^, ^33^, ^52^, ^54^, ^60^, ^62^, ^63^, ^64^ as well as policy changes that restricted migrant welfare benefits.^23^, ^25^, ^28^, ^29^, ^30^, ^36^, ^37^, ^38^, ^39^, ^41^, ^43^, ^44^, ^49^, ^50^, ^51^, ^56^, ^65^, ^66^

Our review has several strengths, including its systematic and comprehensive approach to synthesising all available evidence from the scientific peer-reviewed literature.^14^ We also considered the adequacy of the counterfactuals or comparators used to estimate policy effects.^70^, ^71^ However, our decision to exclude grey literature and restrict our search languages by our linguistic competencies might have excluded some relevant policy evaluations, particularly those outside of the European or North American contexts. The certainty of evidence was largely affected by our reliance on observational studies which often do not employ more advanced analytical strategies found in natural experiment designs to identify policy effects.^70^, ^71^ The heterogeneity of migrant populations and policies, including whether policy introductions or withdrawals were assessed,^72^ and the heterogeneity in comparators, health outcomes, country contexts, and study designs, constrained our ability to do meta-analyses. Inadequate reporting of policy implementation^73^ and statistical results further limited potential narrative synthesis. Despite these challenges, we were able to synthesise and meta-analyse findings related to entry and integration policies.^74^ However, evidence was sparse for exit policies. Additionally, no included studies assessed return or circular migration, preventing assessment of these migration phases.^75^ Other frameworks, such as the lifecourse perspective,^76^ could also have been used to structure the review or provide alternative insights into our findings.

The Edinburgh Declaration on Migration, Ethnicity, Race, and Health^77^ has advocated for more empirical evidence to understand and tackle inequalities. Our review reveals that non-health-targeted policies contribute to the production of health inequalities among migrants. Given the rapid nature of policy change, future research should aim to update the work summarised here to maintain an up-to-date evidence base on the health effects of public policy in migrants. Substantial study design limitations persist, with shortages of natural experiment studies that use robust analytical approaches to isolate policy effects (eg, difference-in-difference analyses with appropriate counterfactuals); of low-income and middle-income study contexts and international comparisons of such policies; and of health research on resettlement, short-term integration, and voluntary or involuntary return policies. These gaps should also be addressed in future research efforts (panel).

Panel **Recommendations for future research**

**Future research directions**

• Research should evaluate the effects of non-health-targeted policies on migrant health in low-income and middle-income countries, as the existing evidence base is derived from high-income countries.
• More studies focusing on the health effects of resettlement (dispersal), short-term integration (language training), and involuntary return (deportation) policies are needed.
• The differential health impacts of policies targeted at the social determinants of health, on migrants compared with non-migrants, remain poorly investigated; these include major macroeconomic, education, employment, social security, and housing policies which might be expected to affect migrants more than native populations.
• Consideration of the medium-term and long-term health effects of policies, as well as whether effects differ amongst migrant subgroups, including by gender, age, socioeconomic position, and reason for migration, is needed.
• Studies should evaluate the health effects of potentially desirable policies oriented towards anti-discrimination, citizenship acquisition, or democratic participation; such studies could inform the implementation of compensatory government policies.
• Further research on entry policies is needed for non-refugee migrants in relation to visa and selection criteria to understand their long-term health effects and how such policies might impact health selection.

**Methodological considerations**

• Studies should attempt to consider how best to create a counterfactual for assessment of causal effects.
• Adequate reporting of policy implementation and its context is essential to ensure results can be interpreted meaningfully.
• Further efforts should be made to theorise the potential mechanisms at play between policies and the specific health outcomes under consideration—when possible, empirical analysis should investigate these mechanisms.
• Data linkage might provide new opportunities for studying the health of migrants in a robust and efficient way, but has been underused thus far.

The findings of this systematic review and meta-analysis provide empirical evidence that non-health-specific public policies can affect migrant health, supporting the importance not only of adopting a Health in All Policies paradigm, but ultimately embracing a human-rights framework that draws attention to the rights of migrants under international law.^78^, ^79^ The human-rights framework considers health as an interdependent human right, whereby action to support some rights could reinforce others—eg, action to provide secure shelter is an important component of ensuring the right to health. Restrictive migration policies (through detention, reduced access to welfare, and so on), therefore, do not only appear to cause health harms, as our findings show, but fundamentally undermine human rights more broadly. Although international law provides a supportive institutional context for improving the health of migrants, its enforcement to meet human-rights obligations is weak. Achieving healthy migration policies requires an appreciation of the conflicting policy interests, with often hostile domestic views on migration being weighed up against potential improvements to the health and human rights of migrants.^80^, ^81^ While improving the evidence base is an important part of improving migrants' health, political action is also ultimately required.
