# Supplementary material for: Effects of non-health-targeted policies on migrant health: a systematic review and meta-analysis
Source: Lancet Glob Health. 2019 Mar 6;7(4):e420–35. doi: 10.1016/S2214-109X(18)30560-6 (PMC6418177; doi:10.1016/S2214-109X(18)30560-6)
Supplement: Supplementary appendix [file mmc1.pdf]

THE LANCET
Global Health

Supplementary appendix

This appendix formed part of the original submission and has been peer reviewed. We post it as supplied by the authors.

Supplement to: Juárez SP, Honkaniemi H, Dunlavy AC, et al. Effects of non-health-targeted policies on migrant health: a systematic review and meta-analysis. *Lancet Glob Health* 2019; published online March 6. http://dx.doi.org/10.1016/S2214-109X(18)30560-6.

Effects of non-health-targeted policies on migrant health: a systematic review and meta-analysis

Sol Pía Juárez, Helena Honkaniemi, Andrea C Dunlavy, Robert W Aldridge, Mauricio L Barreto, Srinivasa Vittal Katikireddi*, Mikael Rostila*

*These authors contributed equally

Department of Public Health Sciences, Stockholm University, Stockholm, Sweden (S P Juárez PhD, H Honkaniemi MSc, A C Dunlavy PhD, Prof M Rostila PhD); Centre for Health Equity Studies (CHESS), Stockholm University/Karolinska Institutet, Stockholm, Sweden (S P Juárez PhD, H Honkaniemi MSc, A C Dunlavy PhD, Prof M Rostila PhD); Centre for Public Health Data Science, Institute of Health Informatics, University College London, London, UK (R W Aldridge PhD); The Farr Institute of Health Informatics Research, University College London, London, UK (R W Aldridge PhD); Centre for Data and Knowledge Integration for Health (CIDACS), Fiocruz, Salvador, Brazil (Prof M L Barreto MD); and MRC/CSO Social and Public Health Sciences Unit, University of Glasgow, Glasgow, UK (S V Katikireddi PhD)

Correspondence to:
Helena Honkaniemi
Department of Public Health Sciences, Stockholm University,
106 91 Stockholm, Sweden
helena.honkaniemi@su.se

Contents

Appendix 1. Description of Search Strategy and Example Search String 2
Appendix 2. Effective Public Health Practice Project (EPHPP) Quality Assessment Tool: Modified Version . 3
Appendix 3. Detailed Descriptives 7
Appendix 4. Risk of Bias Assessments 22
Appendix 5. Narrative Synthesis 26
References (Included Studies) 27

Appendix 1. Description of Search Strategy and Example Search String

We used controlled vocabulary when appropriate (e.g., MeSH terms for MEDLINE) and employed a tripartite structure addressing the population (e.g., migrants, refugees), intervention (e.g., detention policy, integration policy), and outcome (e.g., health, mortality, morbidity). These were evaluated using the Peer Review of Electronic Search Strategies (PRESS) 2015 Evidence-Based Checklist.

PubMed:

(((Emigrants and Immigrants[Mesh]) OR (Emigration and Immigration[Mesh]) OR (Transients and Migrants[Mesh]) OR (Refugees[Mesh]))

OR

((country of birth[Title/Abstract]) OR (countries of birth[Title/Abstract]) OR (country of origin[Title/Abstract]) OR (countries of origin[Title/Abstract]) OR (foreign background*[Title/Abstract]) OR (foreign-born[Title/Abstract]) OR (foreign born[Title/Abstract]) OR (foreignborn[Title/Abstract]) OR (foreigner*[Title/Abstract]) OR (expat*[Title/Abstract]) OR (migrant*[Title/Abstract]) OR (immigra*[Title/Abstract]) OR (emigra*[Title/Abstract]) OR (migrate*[Title/Abstract]) OR (migration*[Title/Abstract]) OR (asylum*[Title/Abstract]) OR (refugee*[Title/Abstract]) OR (displaced child*[Title/Abstract]) OR (displaced individual*[Title/Abstract]) OR (displaced people*[Title/Abstract]) OR (displaced person*[Title/Abstract]) OR (displaced population*[Title/Abstract]) OR (displaced women*[Title/Abstract]) OR (evacuated[Title/Abstract]) OR (evacuation*[Title/Abstract]) OR (evacuee*[Title/Abstract]) OR (international adopt*[Title/Abstract]) OR (internationally adopt*[Title/Abstract])))

AND

(((Public Policy[Mesh]) OR (Jurisprudence[Mesh]) OR (Law Enforcement[Mesh]))

OR

((policy*[Title/Abstract]) OR (policies*[Title/Abstract]) OR (politic*[Title/Abstract]) OR (law[Title/Abstract]) OR (laws*[Title/Abstract]) OR (legal*[Title/Abstract]) OR (legislat*[Title/Abstract]) OR (ruling*[Title/Abstract]) OR (enforcement*[Title/Abstract]) OR (reform*[Title/Abstract]) OR (vett*[Title/Abstract]) OR (residenc*[Title/Abstract]) OR (document*[Title/Abstract]) OR (naturali*[Title/Abstract]) OR (citizen*[Title/Abstract]) OR (settlement*[Title/Abstract]) OR (integrated[Title/Abstract]) OR (integration*[Title/Abstract]) OR (dispers*[Title/Abstract]) OR (displacement*[Title/Abstract]) OR (relocated[Title/Abstract]) OR (relocation*[Title/Abstract]) OR (resettled[Title/Abstract]) OR (resettlement*[Title/Abstract]) OR (resettling[Title/Abstract]) OR (detain*[Title/Abstract]) OR (detention*[Title/Abstract]) OR (deport*[Title/Abstract])))

AND

(((Health[Mesh]) OR (Health Status[Mesh]) OR (Diagnostic Self Evaluation[Mesh]) OR (Diseases Category[Mesh]) OR (Morbidity[Mesh]) OR (Mortality[Mesh]) OR (Health Services[Mesh]) OR (Delivery of Health Care[Mesh]) OR (Patient Care[Mesh]))

OR

((health[Title/Abstract]) OR (self-rated health[Title/Abstract]) OR (well-being[Title/Abstract]) OR (well being[Title/Abstract]) OR (wellbeing[Title/Abstract]) OR (disease*[Title/Abstract]) OR (disorder*[Title/Abstract]) OR (morbidit*[Title/Abstract]) OR (mortalit*[Title/Abstract]) OR (psychol*[Title/Abstract]) OR (psychosoc*[Title/Abstract]) OR (risk behavio*[Title/Abstract]) OR (risky behavio*[Title/Abstract]) OR (healthcare[Title/Abstract]) OR (health-care[Title/Abstract]) OR (prescri*[Title/Abstract])))

Appendix 2. Effective Public Health Practice Project (EPHPP) Quality Assessment Tool: Modified Version

Component Ratings

A. Selection Bias

1. Are the individuals selected to participate in the study likely to be representative of the target population?
   a) Very likely
   b) Somewhat likely
   c) Not likely
   d) Can't tell

2. What percentage of selected individuals agreed to participate?
   a) 80-100% agreement
   b) 60-79% agreement
   c) Less than 60% agreement
   d) Not applicable
   e) Can't tell

   Rate this Section (see dictionary): Strong Moderate Weak

B. Study Design

1. Indicate the study design:
   a) Randomized controlled trial
   b) Controlled clinical trial
   c) Cohort analytic (two group pre + post)
   d) Cross-sectional
   e) Case-control
   f) Cohort (one group pre + post (before and after))
   g) Interrupted time series
   h) Controlled before-after
   i) Uncontrolled before-after
   j) Other specify ______________________________
   k) Can't tell

2. Was the study described as randomized? If NO, go to Component C.
   a) No
   b) Yes

3. If Yes, was the method of randomization described? (See dictionary)
   a) No
   b) Yes

4. If Yes, was the method appropriate? (See dictionary)
   a) No
   b) Yes

   Rate this Section (see dictionary): Strong Moderate Weak

C. Confounders

1. Were there important differences between groups prior to the intervention?
   a) Yes
   b) No
   c) Can't tell

The following are examples of confounders:

Race
Sex
Marital status/family
Age
SES (income or class)
Education
Health status
Pre-intervention score on outcome measure

2. If yes, indicate the percentage of relevant confounders that were controlled (either in the design (e.g. stratification, matching) or analysis)?
   a) 80-100% (most)
   b) 60-79% (some)
   c) Less than 60% (few or none)
   d) Can’t tell

   Rate this Section (see dictionary): Strong Moderate Weak

D. Blinding

1. Is the outcome likely to be affected by blinding?
   a) Yes
   b) No
   c) Can’t tell

2. Was (were) the outcome assessor(s) aware of the intervention or exposure status of participants?
   a) Yes
   b) No
   c) Can’t tell

3. Were the study participants aware of the research question?
   a) Yes
   b) No
   c) Can’t tell

   Rate this Section (see dictionary): Strong Moderate Weak

E. Data Collection Methods

1. Were data collection tools shown to be valid?
   a) Yes
   b) No
   c) Can’t tell

2. Were data collection tools shown to be reliable?
   a) Yes
   b) No
   c) Can’t tell

   Rate this Section (see dictionary): Strong Moderate Weak

F. Withdrawals and Drop-Outs

1. Were withdrawals and drop-outs reported in terms of numbers and/or reasons per group?
   a) Yes
   b) No
   c) Can’t tell
   d) Not applicable (i.e. one time surveys or interviews)

2. Indicate the percentage of participants completing the study (if it differs by groups, record the lowest).
    a) 80-100%
    b) 60-79%
    c) Less than 60%
    d) Can’t tell
    e) Not applicable (i.e. retrospective case-control)

    Rate this Section (see dictionary): Strong Moderate Weak

G. Intervention Integrity

1. What percentage of participants received the allocated intervention or exposure of interest?
    a) 80-100%
    b) 60-79%
    c) Less than 60%
    d) Can’t tell

2. Was the consistency of the intervention measured?
    a) Yes
    b) No
    c) Can’t tell

3. Is it likely that subjects received an unintended intervention (contamination or co-intervention) that may influence the results?
    a) Yes
    b) No
    c) Can’t tell

    Rate this Section (see dictionary): Strong Moderate Weak

H. Analyses

1. Are the statistical methods appropriate for the study design?
    a) Yes
    b) No
    c) Can’t tell
2. Did the authors conduct additional analyses to test the robustness of the findings?
    a) Yes
    b) No
    c) Can’t tell

    Rate this Section (see dictionary): Strong Moderate Weak

Global Rating

Please transcribe the information from the component ratings onto this page. See dictionary on how to rate this section.

Selection Bias	1	2	3	N/A
Study Design	1	2	3	N/A
Confounders	1	2	3	N/A
Blinding	1	2	3	N/A
Data Collection Methods	1	2	3	N/A
Withdrawals and Drop-Outs	1	2	3	N/A
Intervention Integrity	1	2	3	N/A
Analyses	1	2	3	N/A

Global Rating for this Paper (circle one):

1 STRONG (no WEAK ratings)
2 MODERATE (one WEAK rating)
3 WEAK (two or more WEAK ratings)

With both reviewers discussing the ratings:

Is there a discrepancy between the two reviewers with respect to the component (A-H) ratings?

a) No
b) Yes

If yes, indicate the reason for the discrepancy

a) Oversight
b) Differences in interpretation of criteria
c) Differences in interpretation of study

Final decision of both reviewers (circle one):
1 STRONG
2 MODERATE
3 WEAK

Appendix 3. Detailed Descriptives

Authors, Year	Country	Data Source	Data Period	Study Design	Population Group	Sampling Frame and Design	Policy Sector	Policy/ Intervention Description	Health Outcome	Mechanism	Other Notes
Amuedo-Dorantes et al., 2013[1]	USA	Unnamed survey	July 2009-Aug 2010	Cross-sectional	Un-documented, deported or intentionally returned Mexican migrants aged 18+ years	*Multistage probability sampling*<br><br>"The survey uses… random selection of geographic units (i.e., sampling sites and points) and time units (i.e., days and eight-hour long sampling shifts) to recruit representative samples of the different Mexican migrant flows traveling through the San Diego-Tijuana border region. Sampling sites... included the Tijuana International Airport, Tijuana Central bus station, and San Ysidro deportation station at the… border." [*Article*]	**Integration (document.)**	**E-Verify** (1996)<br>Online system matching employment eligibility form (I-9) to data from US government records; originally adapted from Illegal Immigration Reform and Immigrant Responsibility Act (IIRAIRA) of 1996	Difficulties obtaining healthcare services	Policy creates fear of using public services such as healthcare due to possible deportation.	
Anderson & Finch, 2014[2]	USA	Behavioral Risk Factor Surveillance System (BRFSS)	Jan-Dec 2009 (Pre-policy)<br><br>May 2010-Apr 2011 (Post-policy)	Controlled before-after	Latino & white Arizona population aged 18-99 years (Language as proxy for acculturation/ migration status)	*Stratified random sampling*<br><br>BRFSS – national telephone survey with stratified random sampling of non-institutionalized US adult population, representative at national/state levels<br><br>Current analysis limited to Arizona respondents	**Integration (document.)**	**Arizona's Support Our Law Enforcement & Safe Neighborhoods Act** (SB 1070; 2010, repealed in 2012)<br>Enforced requirement to carry migration documentation at all times; established state law enforcement responsibility to require documentation during 'lawful contacts'; criminalized work for illegal migrants	Self-rated health	Political climate can increase exposure to social sources of stress (social stress theory) – might be stronger for sub-group of Spanish-speaking Latinos, who are less likely to be acculturated (group position theory).	Same individuals not assessed pre- and post-policy. Natural experiment control group results not presented.
Angus & DeVoe, 2010[3]	USA	Oregon's Public Health Division family planning waiver service data	May 2005-Apr 2008 (18 months pre- & post-Deficit Reduction Act)	Controlled before-after	Oregon's Family Planning Expansion Project users	*Direct element sampling*<br><br>From service use data	**Integration (welfare)**	**Deficit Reduction Act** (DRA; 2005)<br>Required states to collect proof of citizenship to qualify or reapply for federal matching funds for services provided to Medicaid recipients	Visits to family planning services<br><br>Incl. cervical cancer screen., physicals, counselling & education re. birth control & reproductive health	Citizenship documentation requirements are burdensome for patients and care providers, reduce access to family planning and preventive measures.	"Oregon's Family Planning Expansion Project represents an interesting case study of the impact of citizenship documentation regulations because of the relative ease of enrolment in the state's program prior to 2006." [*Article*]

Beniflah et al., 2013[4]	USA	Paediatric emergency department (PED) charts	2009-10 (Pre-policy)<br><br>2011 (Post-policy)	Controlled before-after	Paediatric patients at 2 PEDs in Georgia (Ethnicity as proxy for document-status)	*Direct element sampling*<br><br>Retrospective review of PED electronic medical records	**Integration (document.)**	**Georgia House Bill 87** (HB87; 2011) Granted local law enforcement the authority to enforce immigration laws	% PED visits<br><br>% High acuity PED visits<br><br>Admission rates (for late presentation)	Policy creates fear of using public services such as healthcare due to concerns over exposure of migrant status.	
Borjas, 2003[5]	USA	March Supplement or Annual Demographic Survey (ADS; pre-2003) / Annual Social & Economic Supplement (ASEC; post-2003) of Current Population Survey (CPS)	1995-2001 (Survey years contain info on 1994-2000)<br><br>Pooled: 1995-96 (Pre-policy) 1999-2001 (Post-policy)	Controlled before-after	CPS survey participants aged <65 years, living in private households, classified as natives or immigrants (naturalized citizens or non-citizens) based on status of household head	*Multistage stratified probability sampling*<br><br>CPS March sample includes main CPS sample and additional sample of Hispanic households<br><br>Multi-stage stratified samples with one primary sample unit (PSU) sampled per stratum, where probability of selection is proportional to PSU's population [*cps.ipums.org/cps/sample_designs.shtml*]<br><br>Used population-level sampling weights [*Article*]	**Integration (welfare)**	**Personal Responsibility & Work Opportunity Reconciliation Act** (PRWORA or "Welfare Reform"; 1996) Modified general welfare eligibility rules, prohibited states from using federal funds for newly unqualified migrant recipients (although many states responded with state-funded programs). Limited access to public assistance for migrants arriving after Aug '96, for first 5 years of settlement, including changes to public health insurance coverage for non-emergency services. Ended federal guarantee of income support to poor families with children, with lifetime limit on public assistance, allowed states to tie welfare decisions into reproductive/marital criteria.	Health insurance coverage	Policy creates fear and administrative obstacles for enrolment; "spill-over" effects among migrants who are eligible. However, overall health insurance coverage rates remain stable as migrants seek out other sources of coverage (e.g., via employment).	"To simplify the presentation of the evidence, I classify all persons in the household as foreign-born or native-born based solely on the birthplace of the household head. Similarly, all foreign-born persons in the household will be classified as citizen or non-citizen based on the naturalization status of the household head." [*Article*]<br><br>Citizen children may be classified as non-citizens if their parents are not citizens.
Borrell et al., 2015[6]	18 European countries [*See article*]	European Social Survey (ESS)	2012	Cross-sectional	Immigrants from low-income countries (not included in IMF list of advanced economies), aged >15 years, in private households	*Stratified random sampling*<br><br>Sampling frames of individuals, households and addresses used (varies by country) [*europeansocialsurvey.org/methodology/ess_methodology/sampling.html*]	**Integration (general)**	**National immigrant integration policies** (As defined by Migrant Integration Policy Index [MIPEX] score)<br><br>[*See mipex.eu*]	Health by perceived group discrimination including: Self-reported health Depressive symptoms Limitation of activity	Integration method makes migrants more or less vulnerable to effects of discrimination.	Excluded data on second generation immigrants.

Bozorgmehr & Razum, 2015[7]	Germany	German Federal Statistics Office (FSO)	Pooled: 1994-97 (Pre-reforms) 1997-2007 (Post-reform 1) 2007-13 (Post-reform 2)	Controlled before-after	Asylum-seekers and refugees in Germany	*Direct element sampling*<br><br>All available data. "Collected by local authorities at municipality level, reported to the statistics offices at federal state level, aggregated at national level by the FSO and reported as count data." [*Article*]	**Integration (welfare)**	**Asylum-Seekers' Benefits Act** (1993) Waiting time for regular access to welfare services increased from 12 to 36 months (Reform 1; 1997) and to 48 months (Reform 2; 2007) for asylum-seekers and refugees in Germany	Asylum-seeker and refugee health expenditures	Legal restrictions lead to delayed care, increased direct and administrative costs, increased use of acute services, restricted geographical access and increased health needs.	
Cho, 2011[8]	USA	National Center for Health Statistics (NCHS) (Linked birth & infant death cohort files)	Pooled: 1995-96 (Pre-policy) 1999-2002 (Post-policy)	Controlled before-after	Mexican women that are not foreign residents, with <16 years education	*Direct element sampling*<br><br>"The data set includes information on all infants born alive in the 50 states and DC during these years." [*Article*]	**Integration (welfare)**	**Personal Responsibility & Work Opportunity Reconciliation Act** (PRWORA or "Welfare Reform"; 1996)	Infant mortality rate	Decreased health insurance coverage and women's care use, and social stress of changes in family income.	
Fritsch, 2011[9]	USA	March Supplement or Annual Demographic Survey (ADS; pre-2003) / Annual Social & Economic Supplement (ASEC; post-2003) of Current Population Survey (CPS)	Pooled: 2005 (Pre-policy) 2007 (Post-policy)	Controlled before-after	CPS survey participants living in private households, classified as citizens or non-citizens based on individual status	*Multistage stratified probability sampling*<br><br>[*cps.ipums.org/cps/sample_designs.shtml*]	**Integration (welfare)**	**Deficit Reduction Act** (DRA; 2005)	Health insurance coverage	"Previously falsified claims of citizenship to obtain Medicaid coverage were not able to continue… without physical documentation…" & "legal permanent residents did not fully understand … eligibility", so did not renew benefits [*Article*]	
Fuentes-Afflick et al., 2006[10]	USA	Structured interviews	1999-2001	Cross-sectional	Hispanic women in California, Florida, New York who delivered in 7 hospitals	*Non-random consecutive sampling*<br><br>"In the 3 cities, which had a large population of Latinos, we selected those hospitals that had the largest number of deliveries to Hispanic women in the year before initiation of the study." [*Article*]	**Integration (welfare)**	**Personal Responsibility & Work Opportunity Reconciliation Act** (PRWORA; 1996)<br><br>Additionally: "Florida and Texas adopted the federal eligibility criteria, resulting in restricted access to Medicaid. California and New York opted to continue provided services to ineligible groups using state sources of funding."	Prenatal care use	"Loss of eligibility for public insurance … [and] climate of fear and intimidation among immigrants … may have deterred women from seeking prenatal care." [*Article*]	Poor counter-factual since double (state and immigration status), unsure where observed effect actually comes from.

Giannoni et al., 2016[11]	23 European countries [*See article*]	Eurostat European Union Statistics on Income & Living Conditions (EU-SILC)<br><br>OECD Health Data<br><br>Eurostat Statistics	2012	Cross-sectional	All EU-SILC respondents (adults residing in Europe; migrants classified as persons born outside Europe)	*Stratified random sampling*<br><br>Best sampling frames available in each National Statistical Institute (NSI) [*ec.europa.eu/eurostat/statistics-explained/index.php/EU_statistics_on_income_and_living_conditions_(EU-SILC)_methodology_%E2%80%93_sampling*]	**Integration (general)**	**National immigrant integration policies** (As defined by Migrant Integration Policy Index [MIPEX] score)	Self-assessed poor health<br><br>Self-reported limiting long-standing illness<br><br>Self-reported chronic illness	Poor integration policy can offset the "healthy migrant effect".	"We check if problems in policies for migrant integration at country level influence non-European born or non-European citizens' health differently than local citizens' health." [*Article*]
Goosen et al., 2014[12]	Nether-lands	Community Health Services for Asylum Seekers (MOA) in Netherlands (E-medical records database)	Jan 2000-Dec 2008	Cohort	Accompanied asylum-seeker children	*Direct element sampling*<br><br>"Data were extracted from a unique electronic registration system that contains nationwide longitudinal demo-graphic and reception data of COA, as well as health care data." [*Article*]	**Entry**	**Annual relocation rate between asylum-seeker centres** Relocation mostly for administrative reasons, e.g., opening/closing of asylum-seeker centres and policy of keeping occupancy rates high; sometimes carried out on request (e.g., for family) or for health reasons (e.g., proximity of specialized hospital)	Mental distress	"Previous studies were based on cross-sectional data… measure[d] total number of relocations… [and] may actually reflect the effect of high annual relocation rates in the first years of residence" [*Article*]	
Hatzenbuehler et al., 2017[13]	USA	Behavioral Risk Factor Surveillance System (BRFSS)<br><br>WestLaw & LexisNexis Legal Databases, online material, expert interviews<br><br>Cooperative Congress. Election Survey	2012	Cross-sectional	BRFSS respondents aged 18+ years in 31 US states	*Multistage probability sampling*<br><br>BRFSS respondents selected through random-digit-dialling<br><br>States selected if: "they exhibited significant legislative activity (a minimum of three relevant laws to maximize variability); they had either a large or rapidly growing Latino population in the state (at least 9% Latino or growth in Latino population of at least 75% between 2000 and 2010); and a sufficient number of Latino respondents in the state in the BRFSS dataset." [*Article*]	**Integration (document.)**	**State-level anti-immigrant policies** (E.g., with regards to mobility, labour, post-secondary education, health, other services, language, omnibus)	Mental health: Poor mental health days per month Psychological distress	"Some pathways [between structural factors and mental health] are likely direct and concern access to material resources… In addition… there are psychosocial mechanisms through which social policies that signal social exclusion may impact the mental health of stigmatized populations." [*Article*]	

Ikram et al., 2015[14]	Nether-lands, France, Denmark	Migrant Ethnic Health Observatory (MEHO) Project<br><br>Netherlands & Denmark (linked pop. register & mortality data)<br><br>France (unlinked data from national mortality register)	Nether-lands (1996-2006)<br><br>France (2005-07)<br><br>Denmark (1992-2001)	Cohort	Turkish- and Moroccan-born migrants in Nether-lands, France and Denmark (compared to native-born samples)	*Direct element sampling*<br><br>Netherlands & Denmark (National population register)<br><br>France (National mortality register)	**Integration (general)**	**National immigrant integration policies**<br>1. **Multicultural model** (Netherlands) – "political and social tolerance and respect of cultural differences with facilities to acquire the citizenship through residence"<br>2. **Differential exclusionist model** (Germany) – "conjectural presence of immigrants based on the labour market needs"<br>3. **Assimilationist model** (France) – citizenship through residence or place of birth "but is not keen on public manifestations of cultural differences"	Mortality:<br>All-cause<br>Suicide<br>Homicide<br>Cardio-vascular disease<br>Respiratory diseases<br>Infectious diseases<br>Cancer<br>Unintentional injuries<br>Other causes	"Mortality would be greater for causes of death that are more sensitive to material and psychosocial conditions and larger socio-economic inequalities in mortality."<br><br>"Partly reflected the pattern in the local-born population, suggesting a shared exposure to adverse environmental factors." [*Article*]	"We should acknowledge that differential exposures in the host country other than the policy environment might also explain the mortality patterns observed in this study."<br><br>"Differences in healthcare access are another factor… In the three countries we studied, legally registered immigrants have the same rights in access to health-care as… nationals." [*Article*]
Johnston et al., 2009[15]	Australia	Surveys (interviews)	2004-05	Cross-sectional	Adult Iraqi refugees with Temporary Protection Visas (TPV) & Permanent Humanitarian Visas (PHV) who arrived in Australia in 1999 or later	*Non-probabilistic sampling / Snowballing*<br><br>Recruited through community and non-gov't organizations with aim to represent various sociodemographic factors	**Entry**	**Temporary Protection Visa Policy** (TPV Policy; 1999-2008) Introduced distinction between TPVs and PPVs (same population, different entry modes):<br>1. **TPV** – excluded/ limited from gov't-funded benefits and services (full income support, free English language tuition, gov't supported university places, interpreting and settlement services) and barred from family reunion programs, had mandatory detention from 1992-2008<br>2. **Permanent protection visas (PPV)** granted to persons entering via offshore humanitarian program, guaranteed immediate refugee status and entitlement to all services provided to permanent residents	Access to healthcare/ medications<br><br>General/ physical health<br><br>Psychological health (distress and depression)<br><br>Personal wellbeing	"While surveyed TPV refugees reported significantly more difficulties in accessing education, English language tuition and welfare, compared with PHV refugees, their narratives were more focused on the pervasive and detrimental impact of the interminable uncertainty about their future, social isolation, anger and sense of injustice." [*Article*]	Mixed-methods - only reported quantitative findings here.

Joyce et al., 2001[16]	USA	Birth files	1995 (Pre-policy)<br><br>1998 (Post-policy)	Cohort analytic (two groups pre & post)	Resident Latinas (US- vs. foreign-born) in California, New York City, Texas who delivered a live infant in 1995 or 1998	*Direct element sampling*<br><br>All relevant birth files in California, NYC, Texas	**Integration (welfare)**	**Personal Responsibility & Work Opportunity Reconciliation Act** (PRWORA or "Welfare Reform"; 1996)<br><br>Additionally: "in Texas... state officials moved swiftly to end financial support for prenatal care among immigrants unqualified for Medicaid under PRWORA... [while in CA & NYC]... state funds for prenatal care were used to replace federal monies lost under PRWORA." [*Article*]	Financing of care (insured vs. uninsured/ self-pay)<br><br>Early initiation of prenatal care<br><br>Proportion of low-birthweight births	"Loss of Medicaid eligibility as well as confusion and fear of scrutiny may have caused immigrants to initiate prenatal care later."<br><br>"No change in early initiation of prenatal care and low birthweight… evidence that PRWORA has had no detectable impact on the perinatal out-comes of foreign-born women." [*Article*]	In the discussion it says that the un-documented population is included but in the introduction that this population was not affected by the policy. We guess it affected their fear of accessing services (i.e., chilling effect). Unable to identify un-documented persons to remove from analysis, combined with other migrants.
Kandula et al, 2004[17]	USA	March Supplement or Annual Demographic Survey (ADS; pre-2003)/ Annual Social & Economic Supplement (ASEC; post-2003) of Current Population Survey (CPS)	1994-2001 (Survey years contain info on 1993-2000)<br><br>Pooled: 1994-96 (Pre-policy) 1998-2001 (Post-policy)	Controlled before-after	Cohorts living in families with income below 200% of federal poverty level:<br><br>1. Pre-1996 migrants (qualified)<br>2. US-born	*Multistage stratified probability sampling*<br><br>CPS March sample includes main CPS sample and additional sample of Hispanic households [*cps.ipums.org/cps/sample_designs.shtml*]<br><br>Used population-level sampling weights [*Article*]	**Integration (welfare)**	**Personal Responsibility & Work Opportunity Reconciliation Act** (PRWORA or "Welfare Reform"; 1996)	Medicaid enrolment	Policy creates fear and administrative obstacles for enrolment; "spill over" effects among migrants who are eligible.	
Kaushal & Kaestner, 2005[18]	USA	March Supplement or Annual Demographic Survey (ADS; pre-2003)/ Annual Social & Economic Supplement (ASEC; post-2003) of Current Population Survey (CPS)	1994-2001 (Survey years contain info on 1993-2000)<br><br>Pooled: 1994-96 (Pre-policy) 1998-2001 (Post-policy)	Controlled before-after	Low-educated, foreign-born single mothers & their children, compared by nativity (vs. US-born), marital status (vs. married), sex (vs. men)	*Multistage stratified probability sampling*<br><br>[*cps.ipums.org/cps/sample_designs.shtml*]	**Integration (welfare)**	Direct effects: **Personal Responsibility & Work Opportunity Reconciliation Act** (PRWORA or "Welfare Reform"; 1996)<br><br>Indirect effects: Denied/limited migrant participation in **Temporary Aid to Needy Families** (TANF; previously Aid to Families with Dependent Children or AFDC), entry point into Medicaid for 5 years	Health insurance coverage	Policy provisions and/or stigma attached to it, such as a "chilling" effect among eligible migrants. Health effects of this could vary, depending on whether migrants would seek out employment with insurance coverage to compensate for this change.	"Heterogeneous responses by states to create substitute [TANF] or Medicaid… are used to investigate whether the estimated effect of PRWORA on newly arrived immigrants is related to the actual provisions of the law, or the result of fears engendered by the law [also known as the "chilling effect"]."<br><br>Included both second- and first-gen children.

Kaushal & Kaestner, 2007[19]	USA	National Health Interview Surveys (NHIS)	Pooled: 1992-96 (Pre-policy) 1998-2002 (Post-policy)	Controlled before-after	Low-educated, foreign-born mothers (18-54 years) and their children (aged 0-14 years); stratified by mothers' marital status	*Multistage probability sampling*<br><br>Continuous representative sampling of households and non-institutional group quarters, with oversampling of minority populations [*cdc.gov/nchs/nhis/about_nhis.htm*]	**Integration (welfare)**	**Personal Responsibility & Work Opportunity Reconciliation Act** (PRWORA or "Welfare Reform"; 1996)	Health insurance coverage<br><br>Medical care utilization<br><br>Self-reported health outcomes	Policy can decrease health insurance coverage & service use, change life circumstances/ opportunities of mothers (welfare-to-work transition, associated stressors).	Included both second- and first-gen children.
Kim & Lee, 2011[20]	South Korea	Biological data on blood lead levels and other lead biomarkers (Soochuyang Institute of Environment. & Occupation. Medicine)	1997 (Pre-policy)<br><br>2005 (Post-policy)	Cross-sectional (data not compared before-after)	Male migrant workers in lead industry	*Not reported*	**Integration (document.)**	**Legal employment permit system for qualified migrants** (2003)<br>Granted unskilled, formerly undocumented migrant workers benefits that "legitimate" workers receive: regular health exam, insurance scheme	4 measures of blood lead levels and other lead biomarkers	Legal working status of migrants should improve health among formerly un-documented workers due to increased workers' rights, including right to health exam.	No info on sampling or extent to which blood lead levels tested among workers, if this was randomly done or not, or why biomarkers were collected on individuals in study.
Korenbrot et al., 2000[21]	USA	Electronic birth certificate database (for California residents)	1990-97 (Years of interest 1994-97)<br><br>1994 (Baseline) 1995 (Post-policy 1) 1996-97 (Post-policy 2)	Controlled before-after	All singleton births from 1990 to 1997 by maternal natality (foreign- and US-born)	*Direct element sampling*<br><br>Not explicitly reported; presumably includes all registered births in CA from 1990-97	**Integration (welfare)**	**California Prop. 187** (1994)<br>Banned state Medicaid coverage of federal-defined non-emergency pregnancy-related services for foreign-born, and emergency services; passed but held up in courts<br><br>**Personal Responsibility & Work Opportunity Reconciliation Act** (PRWORA or "Welfare Reform"; 1996)<br><br>**National migration laws** (1996)<br>Medicaid became potential liability for legal residents wishing to sponsor new migrants, with higher income requirements of sponsors (i.e., Medicaid use as evidence of lack of resources)	Prenatal care coverage<br><br>Use of prenatal care<br><br>Birth outcomes	Passage of policies results in restrictions/ assumed restrictions on access to care, or a culture of fear related to deportation, which will result in poorer birth outcomes.	Both policies were held up in court, which affected actual implementation. Findings may then be related to "chilling effects".<br><br>Also does not really differentiate between effects of one policy vs. another, although estimates from 1995 and 1996 can be "attributed to" 1994 policy Prop. 187.

Levecque & Van Rossem, 2015[22]	20 European countries [*See article*]	European Social Survey (ESS)	2006-07	Cross-sectional	Foreign- and native-born persons aged 15+ years, living in private households in 20 countries in the ESS	*Stratified random sampling*<br><br>Pooled & representative sampling of individuals, households and addresses used (varies by country) [*europeansocialsurvey.org/methodology/ess_methodology/sampling.html*]<br><br>Used population-level sampling weights [*Article*]	**Integration (general)**	**National immigrant integration policies** (As defined by the Migrant Integration Policy Index [MIPEX] score)	Depression	General migrant integration approach in each country may shape mental health effects of being a migrant.	
Loue et al., 2005[23]	USA	Interviews	1999-2001	Cross-sectional	Women of Mexican ethnicity resident in San Diego County, California	*Non-random consecutive sampling*<br><br>“Recruited through churches, clinics, community-based organizations, parent-teacher associations, schools, and social organizations.” [*Article*]	**Integration (welfare)**	**Personal Responsibility & Work Opportunity Reconciliation Act** (PRWORA or "Welfare Reform"; 1996)<br><br>**Illegal Immigration Reform & Immigrant Responsibility Act** (IIRAIRA; 1996) Families need to repay US gov’t for public benefits, including Medicaid, previously received at no cost	Difficulty receiving care<br><br>Satisfaction with healthcare	Reform would affect different migrant groups and access to/quality of care differentially.	
Lurie, 2008[24]	USA	Survey of Income & Program Participation (SIPP)	1996 (Pre-policy)<br><br>2001 (Post-policy)	Controlled before-after	Children of non-citizen permanent (PRs) & non-permanent residents (NPRs) in states with largest migrant pop. (CA, TX, FL, NY, IL, NJ)	*Complex, multistage sampling*<br><br>Primary sample units from Master Address File maintained by US Census Bureau [*census.gov/programs-surveys/sipp/methodology/sampling.html*]	**Integration (welfare)**	**Personal Responsibility & Work Opportunity Reconciliation Act** (PRWORA or "Welfare Reform"; 1996)	Child health insurance status	Non-permanent residents may be afraid to register their children for health insurance coverage even if children are eligible.	Flawed sample – does not distinguish citizen children from non-citizen children (or first- vs. second-generation).
Malmusi, 2015[25]	14 European countries [*See article*]	European Union Survey of Income & Living Conditions (EU-SILC)	2011	Cross-sectional	Individuals aged 16+ years born in country of residence or outside EU, having resided 10+ years in selected country	*Stratified random sampling*<br><br>[*ec.europa.eu/eurostat/statistics-explained/index.php/EU_statistics_on_income_and_living_conditions_(EU-SILC)_methodology_%E2%80%93_sampling*]	**Integration (general)**	**National immigrant integration policies** (As defined by the Migrant Integration Policy Index [MIPEX] score, grouped into typologies by Meuleman & Reeskens)	General self-rated health	Different integration policies may segregate migrants into lower educational/ occupational classes, resulting in discrimination and poor health.	Unlike second Malmusi (2017) paper, compares integration policies to each other.

Malmusi et al., 2017[26]	17 European countries [*See article*]	European Social Survey (ESS)	2012	Cross-sectional	Immigrants from low-income countries (not included in IMF list of advanced economies) & native-born residing in selected countries, aged >15 years and resident in private households	*Stratified random sampling*<br><br>[*europeansocialsurvey.org/methodology/ess_methodology/sampling.html*]<br><br>"For analyses by integration regime, and to account for the different sizes of the countries within integration regimes, this weight was multiplied by a country-level weight and the resulting weight was divided by its average within each group of countries." [*Article*]	**Integration (general)**	**National immigrant integration policies** (As defined by the Migrant Integration Policy Index [MIPEX] score, grouped into typologies by Meuleman & Reeskens)	Depressive symptoms	Inequalities in depression due to poorer living conditions among migrants, which may be worse in countries with more restrictive integration policies (exclusionist).	Compares findings within policies for immigrants vs. non-immigrants (not an appropriate counterfactual since not comparing across integration regimes for migrants alone). Can be compared narratively, however.
Miranda et al., 2011[27]	USA	Hispanic Established Populations for Epidemiol. Studies of Elderly (H-EPESE; baseline)	1993-94	Cross-sectional	Community-dwelling Mexican-origin individuals in Southwestern US 65+ years	*Complex, multistage sampling*<br><br>Recruited from communities [*www.icpsr.umich.edu/icpsrweb/NACDA/studies/25041*]<br><br>Population-level weights	**Entry**	**Sociopolitical context of entry**<br><br>1. **Post-Mexican Revolution** (1918-28) – lenient policy<br>2. **Era of Variable Deportations** (1929-41) – increased deportation due to domestic labour concerns, but variable enforcement of policies<br>3. **The Bracero Era** (1942-64) – Bracero Program employed Mexican migrants on expansion projects<br>4. **Post-Immigration Reform and Control Act** (IRCA; 1965-94) – increased deportation but social protection of Bracero participants	Depressive symptoms	Hostile environment, risk of deportation, as well as protective factors, e.g., labour opportunities, have long-term mental health effects.	
Momartin et al., 2006[28]	Australia	Early Intervention Program of Service for Treatment & Rehabilitation of Torture & Trauma Survivors (STARTTS; Sydney, New South Wales)	2002-03	Cross-sectional	Persian-speaking asylum seekers in Australia granted permanent or temporary protection visas	*Non-random consecutive sampling*<br><br>Recent refugee referrals to STARTTS by resettlement agencies	**Entry**	**Temporary Protection Visa Policy** (TPV Policy; 1999-2008)	Mental health:<br>Post-traumatic stress disorder symptoms<br>Distress<br>Anxiety<br>Depression<br>Mental functional impairment	TPVs and experience of detention, uncertainty regarding settlement experience may result in more negative mental health effects than in PPVs.	Since it was repealed in '08 and all refugees now granted PPVs, can be seen as a proxy measure for the policy change (for instance, thinking of the difference in health outcomes for someone arriving pre-'08 as a TPV and post-'08 as a PPV).

Nam, 2008[29]	USA	March Supplement or Annual Demographic Survey (ADS; pre-2003)/ Annual Social & Economic Supplement (ASEC; post-2003) of Current Population Survey (CPS)	1994-96, 2001-05 (Survey years contain info on 1993-95, 2000-04)<br><br>Pooled: 1994-96 (Pre-policy) 2001-05 (Post-policy)	Controlled before-after	Non-institutional. individuals aged 65+ years (Native Americans excluded)	*Multistage stratified probability sampling*<br><br>[*cps.ipums.org/cps/sample_designs.shtml*]<br><br>“CPS weight variables were created to address the issues of sampling design, nonresponses, and other non-sampling errors.” [*Article*]	**Integration (welfare)**	**Personal Responsibility & Work Opportunity Reconciliation Act** (PRWORA or "Welfare Reform"; 1996)	Elderly health insurance status	Effects of welfare reform on health insurance coverage vary by immigrant citizenship status and length of stay.	Similar dataset as Nam, 2011.
Nam, 2011[30]	USA	March Supplement or Annual Demographic Survey (ADS; pre-2003)/ Annual Social & Economic Supplement (ASEC; post-2003) of Current Population Survey (CPS)<br><br>‘08 Medicaid manuals, e-sources, interviews	1994-96, 2003-08 (Survey years contain info on 1993-95, 2002-07)<br><br>Pooled: 1994-96 (Pre-policy) 2003-08 (Post-policy)	Controlled before-after	Non-institutionalized individuals aged 65+ years (Native Americans excluded)	*Multistage stratified probability sampling*<br><br>[*cps.ipums.org/cps/sample_designs.shtml*]<br><br>“All analyses in this study used weighted data with a weight variable created by CPS that accounted for sampling design and non-response bias.” [*Article*]	**Integration (welfare)**	**Personal Responsibility & Work Opportunity Reconciliation Act** (PRWORA or "Welfare Reform"; 1996)	Elderly health insurance status	Effects of welfare reform on health insurance coverage vary by state generosity, and only for immigrants.	
Pati & Danagoulian, 2008[31]	USA	National Health Interview Surveys (NHIS)	Pooled: 1997-2000 (Pre-policy reversal) 2001-04 (Post-policy reversal)	Controlled before-after	Children living in families with incomes below 200% of federal poverty level	*Multistage probability sampling*<br><br>Continuous sampling “Multistage area probability design that permits the rep-resentative sampling of households and non-institutional group quarters." [*cdc.gov/nchs/nhis/about_nhis.htm*]<br><br>“SAS-callable SUDAAN was used in weighting all of the analyses so that they were nationally representative and in calculating standard errors.” [*Article*]	**Integration (welfare)**	**Illegal Immigration Reform & Immigrant Responsibility Act** (IIRAIRA; 1996)<br><br>Reversal of public charge rule (1999) Medicaid benefits became exempted from the public charge rule of the IIRAIRA	Child health insurance status	Reversal of public charge rule fails to encourage migrants to enrol their children in public health insurance programs, leading to increased rates of uninsured in foreign-born children.	

Patler & Laster Pirtle, 2017[32]	USA	Deferred Action for Childhood Arrivals (DACA) Study (telephone survey)	2014-15	Cross-sectional	Latino DACA-eligible (applied or considered applying) youths in California	*Nonprobability purposive sampling*<br><br>Sample drawn from pool of DACA workshop attendees (in libraries, schools, and convention centres by legal service providers, immigrants' rights organizations, consular offices) between 2012 and 2014	**Integration (document.)**	**Deferred Action for Childhood Arrivals** (DACA; 2012)<br>Two-year (renewable) prosecutorial discretion for unauthorized migrants re. deportation, with work authorization and access to Social Security card; for those who arrived in US before turning 16 years (and have proof) and were aged <31 years when program began on Aug 15, 2012	Psychological wellbeing	Legal status legitimizes recipients and makes them feel more secure, leading to improved psychological wellbeing.	
Reijneveld et al., 2005[33]	Nether-lands	Psychological assessments & interviews	2002-03 (Routine reception)<br><br>2003 (Campus reception)	Cross-sectional	Un-accompanied adolescent asylum seekers (UAASs) in restricted campus ('03) & routine ('02) reception centres, in 6 mo. of arrival	*Nonprobability convenience sampling*<br><br>Campus sample: UAASs eligible if spoke one of 20 languages in questionnaires<br><br>Routine sample: national sample of UAASs	**Entry**	**Unaccompanied adolescent asylum seeker reception policies:**<br><br>1. **Campus reception** (from Nov 2002-Jan 2005) – all UAASs aged 15+ years were located in one campus for minors only with highly structured daily program emphasizing repatriation, with limited possibilities to 'integrate' into Dutch society (e.g., leave campus, learn Dutch), and with constant adult monitoring<br>2. **Routine reception** (up to Nov 2002) – UAASs received in same facilities as adult asylum seekers; allowed opportunities to 'integrate' with Dutch society (e.g., could leave centres during the day, receive guests & attend Dutch language courses)	Mental health	"Uncontrolled immigration has concerned and compelled host countries to enforce stringent restrictions on persons seeking asylum so as to decrease the flow of asylum seekers… May be detrimental for the mental health of asylum seekers… [and] add to the already high prevalence of mental disorders among them... Children and young people seeking asylum are probably particularly sensitive to this." [*Article*]	

Rhodes et al., 2015[34]	USA	North Carolina (NC) Vital Statistics System (vital records collected by NC State Center for Health Service)	Pooled:<br>May 2005-Feb 2006 (Pre-policy)<br>Mar-Dec 2009 (Post-policy)<br><br>Data accessed 2012	Controlled before-after	Mothers identified through NC birth certificate records, including Hispanic/ Latina & non-Hispanic/ Latina mothers	*Direct element sampling*<br><br>Full vital records data	**Integration (document.)**	**Immigration & Nationality Act Section 287(g)** (from IIRAIRA; 1996)<br>Allowed local law enforcement to enforce federal immigration law by targeting un-documented migrants convicted of various crimes (violence, human & narcotics smuggling, gang & organized crime, sexual offenses, & money laundering)<br><br>**Secure Communities Program** (SCP)<br>Facilitated sharing of pertinent info re. local arrestees (fingerprints, ICE data) with FBI, to confirm documentation status via databases	Prenatal care:<br>Late entry into prenatal care<br>Inadequate prenatal care	Policy creates fear of accessing healthcare services.	Authors conducted a mixed-method study. The results of the focus group and in-depth interviews are not reported in the extraction sheet.<br><br>Only mention SCP in beginning, but may not actually assess SCP specifically. SCP was another policy that overlaps with 287(g) to also increase fear of service use.
Salmasi & Pieroni, 2015[35]	Italy	Birth Sample Survey (BSS) (Conducted by Italian Institute of Statistics, ISTAT)	July 2000-June 2001 (Pre-policy; for 2002 survey)<br><br>2003 (Post-policy; for 2005 survey)	Controlled before-after	Mothers born outside Italy vs. selected groups of foreign-born mothers with Italian nationality and Italian mothers	*Sampling design unknown*<br><br>Interviews of 100,000 mothers, including representative sample of foreign-born mothers, both with and without Italian nationality	**Integration (document.)**	**Italian Law 189/2002**<br>Granted amnesty for illegal migrant workers, allowed regularization of migrants in domestic service (expanded to other groups of workers, **Italian Law 222/2002**)<br>650,000 migrants approved (of 705,000 eligible)	Low birthweight	"Reduced… socioeconomic vulnerability… fostered fertility choices… Assumed to promote better use of prenatal care services by immigrant mothers" [*Article*]	
Sommers, 2010[36]	USA	March Supplement or Annual Demographic Survey (ADS; pre-2003)/ Annual Social & Economic Supplement (ASEC; post-2003) of Current Population Survey (CPS)	2004-08 (Survey years contain info on 2003-07)<br><br>Pooled:<br>2004-06 (Pre-policy)<br>2007-08 (Post-policy)	For Medicaid Enrolment: Controlled before-after<br><br>For Medicaid Retention: Cohort analytic (two groups pre & post)	Individuals aged 0-64 years, with focus on non-citizen children (0–18) and adults (19–64), stratified for adults and children	*Multistage stratified probability sampling*<br><br>[*cps.ipums.org/cps/sample_designs.shtml*]<br><br>Weighted with March Supplement of CPS survey weights [*Article*]<br><br>For Medicaid retention, 2-year linked samples from subset of house-holds surveyed in previous year [*Article*]	**Integration (welfare)**	**Deficit Reduction Act** (DRA; 2005)	Medicaid enrolment<br><br>Medicaid retention	Reductions in migrants' enrolment & retention, but may also result in unnecessary administrative costs that outweigh any financial benefits gained from excluding some immigrants from Medicaid.	Self-reported legal status (citizenship), may entail some misrepresentation. Straightforward nature of questions however limits possibility by only asking if respondents are citizens (country of origin also recorded).

Steel et al., 2006[37]	Australia	Surveys (interviews)	Not reported	Cross-sectional	Arabic-speaking Mandaean refugees in Sydney granted permanent or temporary protection visas	*Non-random convenience / Snowballing*<br><br>"Community leaders provided lists… Primary respondents were asked to provide the names and contact details of other Mandaean families." [*Article*]	**Entry**	**Temporary Protection Visa Policy** (TPV Policy; 1999-2008)	Healthcare access<br><br>Mental health: PTSD sympt. Depressive symptoms Mental health status, disability	Past detention and restricted rights of TPVs contribute to PTSD, depression, associated disability in refugees.	Potential for exaggeration bias in reporting.<br><br>Same population treated differently based on their mode of entry.
Steel et al., 2011[38]	Australia	Early Intervention Program of Service for Treatment & Rehabilitation of Torture & Trauma Survivors (STARTTS; Sydney, New South Wales)	2002-03 (Baseline)<br><br>2004-05 (Follow-up)	Cohort analytic (two groups pre & post)	Persian-speaking asylum seekers in Australia granted permanent or temporary protection visas	*Non-random consecutive sampling*<br><br>Recent refugee referrals to STARTTS by resettlement agencies	**Entry**	**Temporary Protection Visa Policy** (TPV Policy; 1999-2008)	Mental health: Post-traumatic stress disorder symptoms Depression Anxiety Distress Excessive worry	Limited access to services and mandatory detention may result in severe mental health consequences in TPVs compared to their PPV counterparts.	"All persons entering the country without valid visas, including asylum seekers, were detained until their refugee status was determined, often a prolonged process spanning several years." [*Article*]
Tan et al., 2016[39]	70 refugee sites in 17 countries in Africa, South & Southeast Asia, Middle East	UN High Commission. for Refugees (UNHCR) Health Info System (HIS)<br><br>UNHCR budget info (FOCUS software)	Jan 2011-Dec 2012	Cross-sectional	Refugees	*Direct element sampling*<br><br>All aggregate data re. refugees under the purview of UNHCR who were resident in UNHCR refugee sites; health data collected through prospective surveillance	**Entry**	**Refugee program spending:** United Nations High Commissioner for Refugees (UNHCR) expenditure on refugee programs in 2011	Crude mortality<br><br>Under-5 mortality	"Particularly dependent and sensitive to the health infra-structure established… by governments, UN agencies such as UNHCR and NGOs." [*Article*]	"This data collection is subject to a number of biases, the most significant being a tendency toward under-reporting of community-based deaths that are not notified to a health facility." [*Article*]
Toomey et al., 2004[40]	USA	Quasi-experimental longitudinal interview study	Mar 2007-Aug 2008 (W1; 3rd trimester of pregnancy)<br><br>Feb 2008-Sept 2009 (W2; child 10 months)<br><br>Apr 2009-Oct 2010 (W3; child 24 months)<br><br>Mar 2010-Dec 2011 (W4; child 36 months)	Other (Open Cohort)	Single, Mexican-origin adolescent mothers (pregnant at recruitment) aged 15-18;<br><br>Their children<br><br>Their mother figures (mother, grandmother, aunt, etc.)	*Unknown*<br><br>Adolescent mothers recruited from schools and community agencies in a large metropolitan city in Arizona	**Integration (document.)**	**Arizona's Support Our Law Enforcement & Safe Neighborhoods Act** (S.B. 1070; 2010, repealed in 2012)	Public assistance<br><br>Preventive health care utilization	Policies that discriminate against immigrant groups can adversely affect their utilization of health care services.	

Urquia et al., 2015[41]	Canada & Spain	**Canada**: Canadian Perinatal Surveillance System, Official immigration register, Citizenship & Immigration Canada database, Discharge Abstracts Database<br><br>**Spain**: Anon birth certificates (National Institute of Statistics)	2000-05 (Canada)<br><br>1998-2007 (Spain)	Cross-sectional	Singleton births during study period for Latin American mothers (and Canadian and Spanish mothers); only birth-weights of 250-5999 g & gestational age within 20-45 weeks	*Direct element sampling*<br><br>All recorded births during study period for populations of interest	**Entry**	**International migrant admission policies:**<br>1. **Canada** (restrictive) – point system rewards characteristics conducive to good labour outcomes (English/French knowledge, education, work experience, wealth); fewer refugees, family-class migrants<br>2. **Spain** (less restrictive) – few refugees, only recently transitioned from emigration to immigration; many unauthorized migrants due to demand for low skill labour, lack of management of migration process	Birth outcomes:<br>Mean birthweight at term<br>Low birthweight<br>Preterm births	Policies may act as first or second filter for self-selection to immigrate. If selective migration is associated with better health we would expect more favourable outcomes among Latin Americans who migrated to Canada compared to those who migrated to Spain, as a result of Canada's stricter selection and admission policies.	
Vargas et al., 2017[42]	USA	Latino National Health & Immigration Survey (LNHIS) (Robert W. Johnson Foundation Centre for Health Policy, Uni. New Mexico)	Jan-Mar 2015	Cross-sectional	Adult Latinos in US (Puerto Rico & 44 states with highest number of Latino residents)	*Complex, multistage sampling*<br><br>Sampled from:<br>1. Cell phone and landline households<br>2. Web surveys (culled from Latino Decisions' national panel of Latino adults, randomly selected to participate in study, weighted to represent Latino pop.)	**Integration (document.)**	**Latino populations' general perceptions of anti-immigration laws:** Including perceptions of state immigrant policy, and then belief of whether or not anti-immigrant or anti-Hispanic sentiments exist	Self-rated health<br><br>Problems with mental health	"Punitive… immigrant laws have led to the perception of 'being hunted' by Immigration and Customs Enforcement, producing intense feelings of anxiety, fear, and depression." [*Article*]	
Venkataramani et al., 2017[43]	USA	National Health Interview Surveys (NHIS)	Pooled:<br>Jan 2008-June 2012 (Pre-policy)<br>June 2012-Dec 2015 (Post-policy)	Controlled before-after	Non-citizen, Hispanic adults aged 19-50 years living in US<br><br>(Excluding individuals with less than high school education & recently arrived immigrants to minimize confounding)	*Multistage probability sampling*<br><br>[*cdc.gov/nchs/nhis/about_nhis.htm*]<br><br>Panel data (annually repeated cross-sectional sample)<br><br>All models use National Health Interview Survey sampling weights [*Article*]	**Integration (document.)**	**Deferred Action for Childhood Arrivals** (DACA; 2012)	Self-rated health<br><br>Psychological distress	DACA increases "employment and income" & "economic opportunities [that] raise future aspirations"; "eliminating the risk of deportation & providing access to employment opportunities could raise hope and reduce psychosocial stress." [*Article*]	Self-reported outcomes, no info on undocumented migrants, institutionalized people not included; "Data for other DACA eligibility criteria - criminal offenses and recent honourable discharge from the military - were also not available in the NHIS" [*Article*]

White et al., 2014[44]	USA	Electronic health record data	Sept 2010-Aug 2012	Interrupted time series	Latino and non-Latino patient visits (adults and children) at 6 community health clinics in Jefferson County, Alabama	*Direct element sampling*<br><br>All health records during study period in Jefferson County, Alabama	**Integration (document.)**	**Alabama Taxpayer & Citizen Protection Act** (House Bill 56; 2011) Required proof of lawful residence to receive state/local benefits including care (including publicly-funded health services) other than those protected by federal law	Visits to healthcare clinics (both for services requiring proof of residence and those exempt from the law)	Proof of residence decreases Latino patient visits more than non-Latino, reflect fear of discovery among undocumented regardless of whether or not services are exempt from law.	Interrupted time series since the data is not pooled.<br><br>"Health department's six community clinics serve as the primary safety net for low-income, uninsured, and underinsured county residents." [*Article*]
Yeo, 2017[45]	USA	National Health Interview Surveys (NHIS)	Pooled: 1993-96 (Pre-policy) 2002-13 (Post-policy)	Controlled before-after	Adults aged 65+ living in US at time of interview	*Multistage probability sampling*<br><br>[*cdc.gov/nchs/nhis/about_nhis.htm*]	**Integration (welfare)**	**Personal Responsibility & Work Opportunity Reconciliation Act** (PRWORA or "Welfare Reform"; 1996)	Outpatient healthcare service use	Welfare reform caused a barrier for indigent immigrant elders to secure health insurance.	Study limitations: NHIS representation issues for elderly, migrants, etc.; migrant arrival year and citizenship status
Zhu & Xu, 2015[46]	USA	March Supplement or Annual Demographic Survey (ADS; pre-2003)/ Annual Social & Economic Supplement (ASEC; post-2003) of Current Population Survey (CPS)	1998-2010	Cross section time series design<br><br>(Pooled state data of native Medicaid coverage & immigrant Medicaid eligibility)	Native- and foreign-born US samples of 1998-2010 March Supplements	*Multistage stratified probability sampling*<br><br>[*cps.ipums.org/cps/sample_designs.shtml*]	**Integration (welfare)**	**Personal Responsibility & Work Opportunity Reconciliation Act** (PRWORA or "Welfare Reform"; 1996)	Inequality in Medicaid coverage	States use restrictive eligibility policies to exclude migrants from safety-net programs, producing negative social constructions	Medicaid coverage gap between migrants and natives larger in states with more exclusive policies, strengthened in states with lower levels of migrant density.

Appendix 4. Risk of Bias Assessments

Authors, Year	Selection Bias Quality	Study Design Quality	Confounder Quality	Blinding Quality	Data Collection Methods Quality	With-drawals & Drop-Outs Quality	Intervention Quality	Analysis Quality	Global Rating	Discrepancy (Reason)	Final Decision	Additional Notes
Amuedo-Dorantes et al., 2013[1]	2	3	2	3	3	2	2	1	3	No	3	Deported and voluntary returnees are not compared to each other, but within each compare E-Verify state-dwelling vs. not
Anderson & Finch, 2014[2]	3	1	1	2	1	2	2	1	2	No	2	Tool validity and reliability: "A systematic review of publications assessing reliability and validity of the Behavioural Risk Factor Surveillance System (BRFSS), 2004-2011"
Angus & DeVoe, 2010[3]	3	2	3	2	2	2	2	3	3	No	3	
Beniflah et al., 2013[4]	2	3	3	2	2	2	3	3	3	No	3	Compared descriptively to changes in Caucasian and African-American patient percentages, but no quantitative comparison (i.e., difference-in-difference analysis).
Borjas, 2003[5]	1	1	1	2	1	2	2	1	1	No	1	Only problem was children and their definition of citizenship.
Borrell et al., 2015[6]	2	2	HH: 1<br>SPJ: 2	2	1	2	2	1	1	No	1	
Bozorgmehr & Razum, 2015[7]	1	1	2	2	1	2	2	1	1	No	1	
Cho, 2011[8]	1	1	1	2	1	2	2	1	1	No	1	
Fritsch, 2011[9]	1	1	2	2	1	2	1	2	1	No	1	
Fuentes-Afflick et al., 2006[10]	1	3	2	2	2	2	2	2	3	No	2	
Giannoni et al., 2016[11]	2	2	1	2	1	2	2	1	1	No	1	
Goosen et al., 2014[12]	HH: 2<br>SPJ: 1	2	3	2	3	3	HH: 2<br>SPJ: 3	2	3	No	3	
Hatzenbuehler et al., 2017[13]	HH: 2<br>SPJ: 1	3	HH: 2<br>SPJ: 3	2	1	2	2	1	HH: 2<br>SPJ: 3	Yes. Adequacy of confounders.	2	
Ikram et al., 2015[14]	1	2	2	2	1	2	2	2	1	No	1	
Johnston et al., 2009[15]	2	3	HH: 2<br>SPJ: 3	3	1	2	HH: 2<br>SPJ: 3	2	3	No	3	
Joyce et al., 2001[16]	2	1	2	2	2	2	HH: 2<br>SPJ: 3	2	2	No	2	
Kandula et al, 2004[17]	ACD: 2<br>HH: 1	2	2	2	1	2	2	1	1	No	1	"For the cases eligible for the 2015 ASEC, the basic CPS household-level nonresponse rate was 13.4 percent. The household-level nonresponse rate for the ASEC was an additional 14.2 percent. These two non-response rates lead to a combined supplement nonresponse rate of 25.7 percent." [*icpsr.umich.edu/icpsrweb/NADAC/studies/36525*]

Kaushal & Kaestner, 2005[18]	ACD: 2<br>HH: 1	2	1	ACD: 1<br>HH: 2	1	2	ACD: 2<br>HH: 3	ACD: 1<br>HH: 2	ACD: 1<br>HH: 2	Authors acknowledge some of the major short-comings in their DD analyses and the lack of appropriate comparison groups.	1	“Nevertheless, the fact that not 100 percent of the target group is affected by changes in the policy will result in downward biased DD estimates of the effect of PRWORA on insurance status (i.e., the effect of treatment on the treated).”<br><br>“In the case of immigrants, the misclassification bias may be larger because we cannot distinguish between permanent legal residents, those affected by PRWORA, and other immigrants (e.g., un-documented immigrants). Therefore, estimates of the effect of PRWORA may differ between natives and immigrants.”<br><br>“It is difficult to assess the validity of the comparison groups.” (But validity tested and confirmed) [*Article*]
Kaushal & Kaestner, 2007[19]	2	2	1	ACD: 1<br>HH: 2	1	2	ACD: 1<br>HH: 3	ACD: 1<br>HH: 2	ACD: 1<br>HH: 2	Authors acknowledge some of the major short-comings in their DD analyses and the lack of appropriate comparison groups.	1	“Unfortunately, the NHIS do not provide data on whether a foreign-born person is citizen for all the years used in this analysis. There is also no information on the legal status of immigrants, which may affect their health insurance and health [42, 43]. Therefore, it is not possible to control for a respondent’s immigrant or citizenship status. This limitation is partly overcome by controlling for the number of years lived in the U.S. since citizenship is highly correlated with duration of residence in the US. The NHIS does not provide a foreign-born person’s country of birth, but it provides detailed information on her ethnicity, which is used to define the following ethnic groups: non-Hispanic black, non-Hispanic white, Hispanic, Asian and others.” [*Article*]<br><br>“The annual response rate of NHIS is approx. 80 percent of the eligible households in the sample.” [*cdc.gov/nchs/nhis/about_nhis.htm*]
Kim & Lee, 2011[20]	3	3	3	1	1	2	2	ACD: 2<br>HH: 3	3	No	3	
Korenbrot et al., 2000[21]	1	2	2	ACD: 1<br>HH: 2	1	ACD: 1<br>HH: 2	3	2	2	No	2	“In this analysis we are unable to distinguish among the foreign-born women who are undocumented, non-permanent residents, permanent residents (qualified or non-qualified) and naturalized citizens. Therefore the size of effects we examine are for all foreign-born women, regardless of their immigrant status.” [*Article*]
Levecque & Van Rossem, 2015[22]	2	2	1	2	1	2	3	2	2	No	2	
Loue et al., 2005[23]	2	3	3	3	2	2	2	3	3	No	3	

Lurie, 2008[24]	HH: 3<br>ACD: 2	HH: 1<br>ACD: 2	1	2	1	2	HH: 3<br>ACD: 2	2	HH: 3<br>ACD: 2	Agreed on 2, because only real problem is citizenship risk.	2	Lack of identification of migrant status among children of migrants is a serious limitation.
Malmusi, 2015[25]	2	HH: 2<br>ACD: 3	2	2	1	2	2	HH: 1<br>ACD: 2	HH: 1<br>ACD: 2	Resolved.	2	
Malmusi et al., 2017[26]	2	2	2	2	1	2	2	2	HH: 1<br>ACD: 2	Wrong comparison group? (Cross-sectional)	2	
Miranda et al., 2011[27]	2	HH: 3<br>ACD: 2	HH: 2<br>ACD: 3	2	1	2	3	2	3	No	3	Difficult to determine extent to which policies implemented within each arrival cohort, affects intervention integrity and appropriateness of the counterfactuals.
Momartin et al., 2006[28]	2	HH: 3<br>ACD: 2	HH: 2<br>ACD: 3	2	1	2	3	2	3	No	3	Issue with separating detention experiences from TPV status.
Nam, 2008[29]	HH: 1<br>ACD: 2	HH: 1<br>ACD: 2	HH: 1<br>ACD: 2	HH: 2<br>ACD: 1	1	HH: 2<br>ACD: 1	HH: 3<br>ACD: 2	HH: 1<br>ACD: 2	HH: 2<br>ACD: 1	Resolved.	2	Not all were exposed to welfare reform effects due to state-wide variations.
Nam, 2011[30]	1	1	HH: 1<br>ACD: 2	HH: 2<br>ACD: 1	1	HH: 2<br>ACD: 1	2	1	1	No	1	
Pati & Danagoulian, 2008[31]	2	2	1	HH: 2<br>ACD: 1	1	2	HH: 3<br>ACD: 2	2	HH: 2<br>ACD: 1	Does not account for state-wide variation in policy.	2	
Patler & Laster Pirtle, 2017[32]	HH: 2 /<br>ACD: 3	2	1	3	2	2	3	HH: 2<br>ACD: 3	3	No	3	Re. Blinding: “We are not attempting to clinically measure psychological conditions; rather, our measures capture more specified health concerns that Latino undocumented immigrants might face. To that end, the DACA Study examined self-reported psychological wellbeing that Latino immigrant youth perceived to be directly impacted by their legal status.” [*Article*]<br><br>PLUS relied on recall for past psychological symptoms.
Reijneveld et al., 2005[33]	HH: 2<br>ACD: 1	HH: 3<br>ACD: 2	HH: 2<br>ACD: 3	HH: 2<br>ACD: 3	1	2	2	HH: 1<br>ACD: 2	HH: 2<br>ACD: 3	Outcome sensitive to blinding, plus poor controls, plus cross-sectional with no randomization/small sample.	3	
Rhodes et al., 2015[34]	2	SPJ: 3<br>ACD: 2	3	2	1	2	2	2	SPJ: 3<br>ACD: 2	Resolved.	2	
Salmasi & Pieroni, 2015[35]	2	2	SPJ: 2<br>ACD: 1	SPJ: 2<br>ACD: 1	1	1	2	1	1	No	1	
Sommers, 2010[36]	SPJ: 2<br>ACD: 1	SPJ: 3<br>ACD: 2	2	SPJ:<br>ACD: 1	2	2	SPJ: 3<br>ACD: 2	1	SPJ: 3 / 1	Resolved.	1	

Steel et al., 2006[37]	3	3	2	3	1	2	2	3	3	No	3	
Steel et al., 2011[38]	3	2	3	SPJ: 1<br>ACD: 2	SPJ: 2<br>ACD: 1	SPJ: 3<br>ACD: 1	SPJ: 1<br>ACD: 2	SPJ: 2 / 3	3	No	3	
Tan et al., 2016[39]	2	3	3	1	2	2	3	1	SPJ/ACD: 2 / HH: 3	Resolved.	3	Minus points for representativeness, cross-sectional design and no controls, plus fact that we are looking at budgets (plans), not actual spending (implementation).
Toomey et al., 2004[40]	3	2	SPJ: 3<br>ACD: 2	2	SPJ/ACD: 2 / HH: 3	3	SPJ: 1<br>ACD: 2	1	SPJ: 3<br>ACD: 2	ACD: 2	3	Rated as 3 based on our quality criteria and the issues of sampling and the non-reporting of drop outs. Plus no reliability/validity for tools, poor controls, etc.
Urquia et al., 2015[41]	1	2	2	1	1	2	3	1	2	No	2	Issue of selection in origin.
Vargas et al., 2017[42]	2	2	2	SPJ: 2<br>ACD: 3	2	2	3	2	SPJ: 2<br>ACD: 3	Resolved.	3	Does not properly assess a policy and the measures are self-reported. Latinos as group designation is limitation; perceptions of hostile policies rather than an actual policy.
Venkataramani et al., 2017[43]	2	SPJ: 3<br>ACD:/HH: 2	2	2 (HH: Not sensitive)	1	2	2	1	SPJ: 2<br>ACD/HH: 1	Resolved.	1	
White, 2014[44]	1	2	3	2	1	1	3	3	3	No	3	
Yeo, 2017[45]	SPJ: 3<br>ACD:/HH: 2	2	3	2	SPJ: 2<br>ACD: 1	2	2	2	SPJ: 3<br>ACD/HH: 2	ACD/HH: 2	2	
Zhu et al., 2015[46]	2	1	1	1	1	2	3	2	SPJ: 3 / ACD/HH: 2	2	2	

Appendix 5. Narrative Synthesis

Policy Context	Health Outcome	Results
**Entry**	Mental health	Studies of refugees granted temporary as opposed to permanent protection visas in Australia demonstrated nearly unanimous evidence of increased symptoms of distress,[15,38] depression and anxiety,[15,37,38] and PTSD.[37,38] Increasingly restricted entry contexts were also affiliated with greater depressive symptoms among Mexican migrants to the US, relative to those who arrived during more generous periods.[27] One exception was the non-significant difference in depression and PTSD symptomology among asylum-seeking adolescents exposed to restricted (versus generous) reception environments.[33]
**Integration** (welfare, documentation)	Self-rated health	One low risk-of-bias analysis of welfare restrictions showed improved SRH among low-educated single migrant mothers relative to their married counterparts.[19] Assessment of documentation policy protecting the rights of undocumented migrants showed no relationship with SRH,[43] although findings with a stronger risk of bias indicated harmful health effects associated with more restrictive documentation policies.[2,42]
**Integration** (documentation)	Other	A South Korean study showed improvements in blood lead levels associated with the documentation-related introduction of legal work permits and access to routine health exams.[20]
**Integration** (welfare)	Public health insurance (i.e., Medicaid) enrolment	A policy that increased waiting times for welfare eligibility revealed little change in enrolment in the general migrant population,[5,17] and for children[5] and the elderly,[29,30] but decreased enrolment among married migrant women.[18] Protective legislation established by some US states seemed to buffer against overall enrolment inequalities between natives and migrants[46] and loss of enrolment among elderly migrants,[29] although differences between protected and unprotected states in insurance enrolment were minimal[5,9] or non-existent.[17,18] Findings of a welfare policy enforcing a burden of proof for eligibility also showed decreased Medicaid enrolment among non-citizens, relative to US citizens.[9,36]
**Integration** (welfare, documentation)	Prenatal care use	Narrative synthesis suggested increased adequacy and early initiation of prenatal care in protected states (e.g., California, New York),[16,21] with other moderate[10,16] and high risk-of-bias evidence[23] indicating no effect of state protection. Unprotected states (e.g., Florida, Texas) exhibited no change[16] or decreases[10] in care use. Related findings on documentation requirements within one US state showed no significant differences in prenatal care use by county, despite prevailing state-wide care deficits amongst Latina versus non-Latina mothers.[34]

References (Included Studies)

1. Amuedo-Dorantes C, Puttitanun T, Martinez-Donate AP. How do tougher immigration measures affect unauthorized immigrants? *Demography* 2013; **50**: 1067–91.
2. Anderson KF, Finch JK. Racially charged legislation and Latino health disparities: the case of Arizona's S.B. 1070. *Sociol Spectr* 2014; **34**: 526–48.
3. Angus L, Devoe J. Evidence that the citizenship mandate curtailed participation in Oregon's Medicaid family planning program. *Health Aff (Millwood)* 2010; **29**: 690–98.
4. Beniflah JD, Little WK, Simon HK, Sturm J. Effects of immigration enforcement legislation on Hispanic pediatric patient visits to the pediatric emergency department. *Clin Pediatr* 2013; **52**: 1122–26.
5. Borjas GJ. Welfare reform, labor supply, and health insurance in the immigrant population. *J Health Econ* 2003; **22**: 933–58.
6. Borrell C, Palencia L, Bartoll X, Ikram U, Malmusi D. Perceived discrimination and health among immigrants in Europe according to national integration policies. *Int J Environ Res Public Health* 2015; **12**: 10687–99.
7. Bozorgmehr K, Razum O. Effect of Restricting Access to Health Care on Health Expenditures among Asylum-Seekers and Refugees: A Quasi-Experimental Study in Germany, 1994–2013. *PLoS One* 2015; **10**: e0131483.
8. Cho RM. Effects of welfare reform policies on Mexican immigrants' infant mortality rates. *Soc Sci Res* 2011; 40: 641–53.
9. Fritsch NT. Immigrants, Medicaid, and the Deficit Reduction Act. *Undergraduate Econ Rev* 2011; **8**: 16.
10. Fuentes-Afflick E, Hessol NA, Bauer T, et al. Use of prenatal care by Hispanic women after welfare reform. *Obstet Gynecol* 2006; **107**: 151–60.
11. Giannoni M, Franzini L, Masiero G. Migrant integration policies and health inequalities in Europe. *BMC Public Health* 2016; **16**: 463.
12. Goosen S, Stronks K, Kunst AE. Frequent relocations between asylum-seeker centres are associated with mental distress in asylum-seeking children: a longitudinal medical record study. *Int J Epidemiol* 2014; **43**: 94–104.
13. Hatzenbuehler ML, Prins SJ, Flake M, et al. Immigration policies and mental health morbidity among Latinos: A state-level analysis. *Soc Sci Med* 2017; **174**: 169–78.
14. Ikram UZ, Malmusi D, Juel K, Rey G, Kunst AE. Association between integration policies and immigrants' mortality: an explorative study across three European countries. *PLoS One* 2015; **10**: e0129916.
15. Johnston V, Allotey P, Mulholland K, Markovic M. Measuring the health impact of human rights violations related to Australian asylum policies and practices: a mixed methods study. *BMC Int Health Hum Rights* 2009; **9**: 1.
16. Joyce T, Bauer T, Minkoff H, Kaestner R. Welfare reform and the perinatal health and health care use of Latino women in California, New York City, and Texas. *Am J Public Health* 2001; **91**: 1857–64.
17. Kandula NR, Grogan CM, Rathouz PJ, Lauderdale DS. The unintended impact of welfare reform on the Medicaid enrollment of eligible immigrants. *Health Serv Res* 2004; **39**: 1509–26.
18. Kaushal N, Kaestner R. Welfare reform and health insurance of immigrants. *Health Serv Res* 2005; **40**: 697–721.
19. Kaushal N, Kaestner R. Welfare reform and health of immigrant women and their children. *J Immigr Minor Health* 2007; **9**: 61–74.
20. Kim NS, Lee BK. A comparison of blood lead levels between migrant and native lead workers before and after implementation of a new employment permit system for migrant workers. *Ind Health* 2011; **49**: 235–41.
21. Korenbrot CC, Dudley RA, Greene JD. Changes in births to foreign-born women after welfare and immigration policy reforms in California. *Matern Child Health J* 2000; **4**: 241–50.
22. Levecque K, Van Rossem R. Depression in Europe: does migrant integration have mental health payoffs? A cross-national comparison of 20 European countries. *Ethn Health* 2015; **20**: 49–65.
23. Loue S, Cooper M, Lloyd LS. Welfare and immigration reform and use of prenatal care among women of Mexican ethnicity in San Diego, California. *J Immigr Health* 2005; **7**: 37–44.

24. Lurie IZ. Welfare reform and the decline in the health-insurance coverage of children of non-permanent residents. *J Health Econ* 2008; **27**: 786–93.
25. Malmusi D. Immigrants' health and health inequality by type of integration policies in European countries. *Eur J Public Health* 2015; **25**: 293–99.
26. Malmusi D, Palencia L, Ikram UZ, Kunst AE, Borrell C. Inequalities by immigrant status in depressive symptoms in Europe: the role of integration policy regimes. *Soc Psychiatry Psychiatr Epidemiol* 2017; **52**: 391–98.
27. Miranda PY, Schulz AJ, Israel BA, Gonzalez HM. Context of entry and number of depressive symptoms in an Mexican-origin immigrant population. *J Immigr Minor Health* 2011; **13**: 706–12.
28. Momartin S, Steel Z, Coello M, Aroche J, Silove DM, Brooks R. A comparison of the mental health of refugees with temporary versus permanent protection visas. *Med J Aust* 2006; **185**: 357–61.
29. Nam Y. Welfare reform and older immigrants' health insurance coverage. *Am J Public Health* 2008; **98**: 2029–34.
30. Nam Y. Welfare reform and elderly immigrants' health insurance coverage: the roles of federal and state Medicaid eligibility rules. *J Gerontol Soc Work* 2011; **54**: 819–36.
31. Pati S, Danagoulian S. Immigrant children's reliance on public health insurance in the wake of immigration reform. *Am J Public Health* 2008; **98**: 2004–10.
32. Patler C, Laster Pirtle W. From undocumented to lawfully present: do changes to legal status impact psychological wellbeing among Latino immigrant young adults? *Soc Sci Med* 2017; **199**: 39–48.
33. Reijneveld SA, de Boer JB, Bean T, Korfker DG. Unaccompanied adolescents seeking asylum: poorer mental health under a restrictive reception. *J Nerv Ment Dis* 2005; **193**: 759–61.
34. Rhodes SD, Mann L, Siman FM, et al. The impact of local immigration enforcement policies on the health of immigrant Hispanics/Latinos in the United States. *Am J Public Health* 2015; **105**: 329–37.
35. Salmasi L, Pieroni L. Immigration policy and birth weight: positive externalities in Italian law. *J Health Econ* 2015; **43**: 128–39.
36. Sommers B. Targeting in Medicaid: The costs and enrolment effects of Medicaid's citizenship documentation requirement. *J Public Econ* 2010; **94**: 174–82.
37. Steel Z, Silove D, Brooks R, Momartin S, Alzuhairi B, Susljik I. Impact of immigration detention and temporary protection on the mental health of refugees. *Br J Psychiatry* 2006; **188**: 58–64.
38. Steel Z, Momartin S, Silove D, Coello M, Aroche J, Tay KW. Two year psychosocial and mental health outcomes for refugees subjected to restrictive or supportive immigration policies. *Soc Sci Med* 2011; **72**: 1149–56.
39. Tan TM, Spiegel P, Haskew C, Greenough PG. Does spending on refugees make a difference? A cross-sectional study of the association between refugee program spending and health outcomes in 70 sites in 17 countries. *Confl Health* 2016; **10**: 28.
40. Toomey RB, Umana-Taylor AJ, Williams DR, Harvey-Mendoza E, Jahromi LB, Updegraff KA. Impact of Arizona's SB 1070 immigration law on utilization of health care and public assistance among Mexican-origin adolescent mothers and their mother figures. *Am J Public Health* 2014; **104**: S28–34.
41. Urquia ML, Vang ZM, Bolumar F. Birth Outcomes of Latin Americans in two countries with contrasting immigration admission policies: Canada and Spain. *PLoS One* 2015; **10**: e0136308.
42. Vargas ED, Sanchez GR, Juarez M. Fear by Association: Perceptions of anti-immigrant policy and health outcomes. *J Health Politics Policy Law* 2017; **42**: 459–83.
43. Venkataramani AS, Shah SJ, O'Brien R, Kawachi I, Tsai AC. Health consequences of the US Deferred Action for Childhood Arrivals (DACA) immigration programme: a quasi-experimental study. *Lancet Public Health* 2017; **2**: e175-e81.
44. White K, Blackburn J, Manzella B, Welty E, Menachemi N. Changes in use of county public health services following implementation of Alabama's immigration law. *J Health Care Poor Underserved* 2014; **25**: 1844–52.
45. Yeo Y. Healthcare inequality issues among immigrant elders after neoliberal welfare reform: empirical findings from the United States. *Eur J Health Econ* 2017; **18**: 547–65.
46. Zhu L, Xu P. The politics of welfare exclusion: immigration and disparity in Medicaid coverage. *Policy Stud J* 2015; **43**: 456–83.
